# Interaction of mitochondrial fission factor with dynamin related protein 1 governs physiological mitochondrial function ***in vivo***

**DOI:** 10.1038/s41598-018-32228-1

**Published:** 2018-09-19

**Authors:** Opher S. Kornfeld, Nir Qvit, Bereketeab Haileselassie, Mehrdad Shamloo, Paolo Bernardi, Daria Mochly-Rosen

**Affiliations:** 1 Department of Chemical and Systems Biology, Stanford University School of Medicine, Stanford, CA 94305 USA; 2 The Azrieli Faculty of Medicine in the Galilee, Bar-Ilan University, Safed, 12325 Israel; 3 Department of Pediatrics, Stanford University School of Medicine, Stanford, CA 94305 USA; 4 Behavioral and Functional Neuroscience Laboratory, Department of Neurosurgery, Stanford University School of Medicine, Stanford, CA 94305 USA; 5 Department of Biomedical Sciences, University of Padova, Padova, 35122 Italy

## Abstract

Mitochondria form a dynamic network governed by a balance between opposing fission and fusion processes. Because excessive mitochondrial fission correlates with numerous pathologies, including neurodegeneration, the mechanism governing fission has become an attractive therapeutic strategy. However, targeting fission is a double-edged sword as physiological fission is necessary for mitochondrial function. Fission is trigged by Drp1 anchoring to adaptors tethered to the outer mitochondrial membrane. We designed peptide P259 that distinguishes physiological from pathological fission by specifically inhibiting Drp1′s interaction with the Mff adaptor. Treatment of cells with P259 elongated mitochondria and disrupted mitochondrial function and motility. Sustained *in vivo* treatment caused a decline in ATP levels and altered mitochondrial structure in the brain, resulting in behavioral deficits in wild-type mice and a shorter lifespan in a mouse model of Huntington’s disease. Therefore, the Mff-Drp1 interaction is critical for physiological mitochondrial fission, motility, and function *in vitro* and *in vivo*. Tools, such as P259, that differentiate physiological from pathological fission will enable the examination of context-dependent roles of Drp1 and the suitability of mitochondrial fission as a target for drug development.

## Introduction

Within a single mammalian cell, thousands of mitochondria form a continuously-changing network. Mitochondrial fission and fusion drive the dynamics of these networks^1^. Mitochondrial fusion mediates complementation of partially damaged mitochondria whereas fission separates healthy from damaged mitochondria, that are subsequently removed by a specific process called mitophagy^2^. An imbalance between mitochondrial fission and fusion leads to dysfunctional mitochondria and is a hallmark of numerous disorders spanning multiple body systems^3,4^. Of importance, excessive fission resulting in fragmented and dysfunctional mitochondria is observed in numerous diseases, including neurodegenerative disorders^5^.

The core machinery responsible for executing mitochondrial fission consists of the cytosolic dynamin related protein 1 (Drp1), a large GTPase that oligomerizes on and constricts the outer mitochondrial membrane^1^. Four outer mitochondrial membrane adaptors anchor Drp1: mitochondrial fission factor (Mff), mitochondrial fission protein 1 (Fis1), and mitochondrial division factors 49 and 51 kDa (MiD49 and MiD51)^1^. We hypothesized that differential recruitment of Drp1 to the outer mitochondrial membrane distinguishes between balanced fission and pathological excessive fission. To study the adaptors’ role in Drp1 recruitment, we sought to develop adaptor-specific inhibitors of Drp1. Our previous work identified a specific Drp1-Fis1 inhibitor, P110, and showed that Fis1 recruits Drp1 under cell stress in numerous neurodegenerative disease models; inhibition of the Fis1-Drp1 interaction has therapeutic benefits in these models^6–10^. Importantly, inhibiting Drp1-Fis1 interaction had no effect on mitochondrial morphology and function under physiological conditions^6^ and was safe when delivered *in vivo* for five months^10^.

However, Drp1 is also required to maintain basal (physiological) fission and inter-organelle interactions^1,5,11,12^. Because cells lacking Mff have more elongated mitochondria than cells lacking any other adaptor^13,14^, we set out to study the physiological role of Drp1 through its interaction with Mff. We designed a novel pharmacological inhibitor of the Drp1-Mff interaction, which we utilized to further elucidate the physiological roles of Drp1. Inhibiting Drp1-Mff interaction *in vitro* did not impede on Drp1′s interactions with its other mitochondrial receptors. In both immortalized and primary neuronal cultures, inhibiting the Mff-Drp1 interaction greatly reduced basal fission. While excessive fragmentation is associated with mitochondrial dysfunction in neurodegenerative disease models, we show that Mff-mediated mitochondrial fission is essential for maintaining mitochondrial activity. Indeed, treatment with this inhibitor resulted in a reduction of brain ATP levels and caused motor and neurological deficits in wild-type mice, demonstrating that inhibiting basal mitochondrial fission alone can cause neuropathology.

## Results

### Rational design of a specific Mff-Drp1 inhibitor

**Figure 1:**
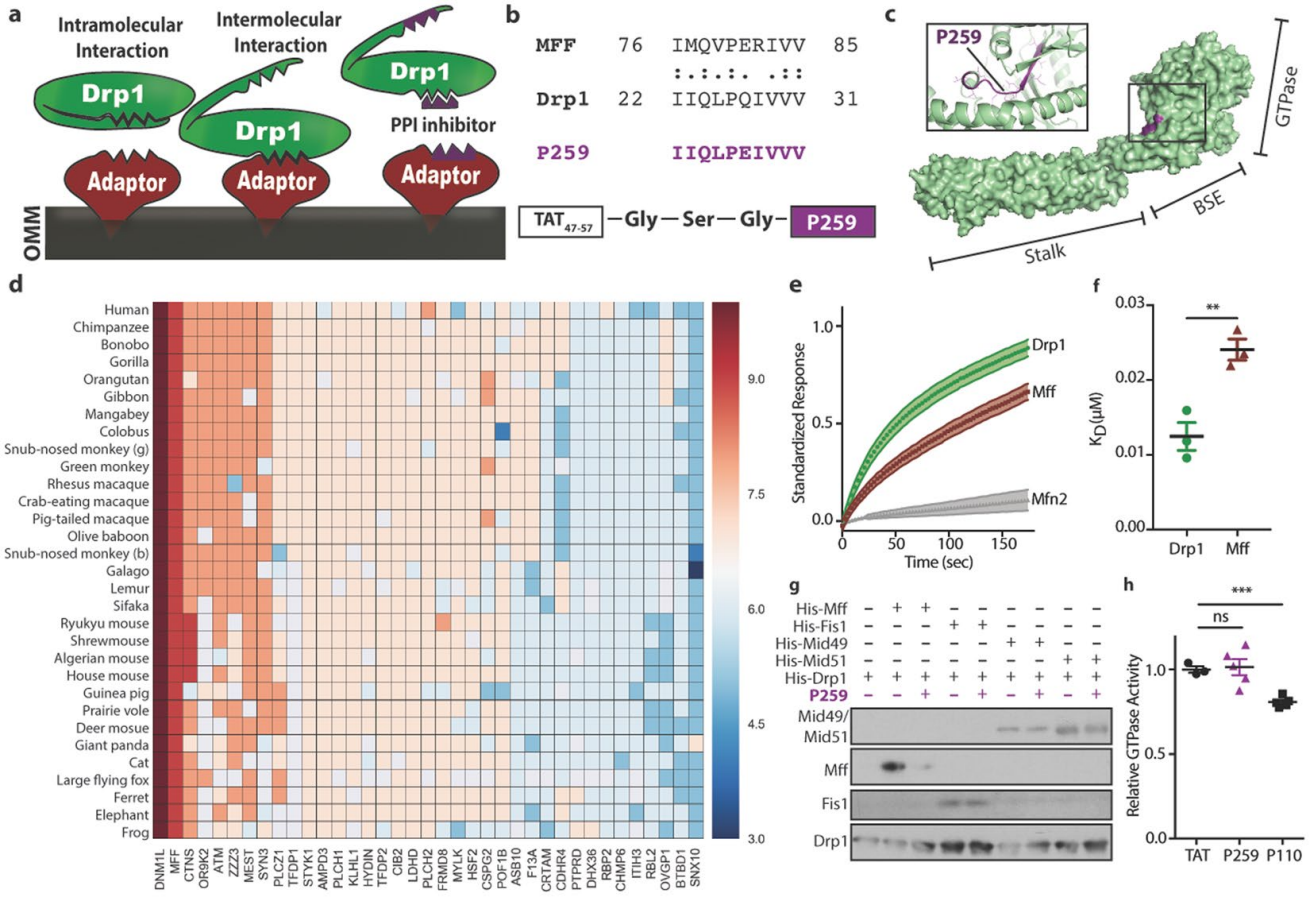
Rational peptide design identifies a novel inhibitor of the Mff-Drp1 protein-protein interaction. **(a)** A scheme of the rationale behind the inhibitor design. (**b**) A short sequence of homology between Mff and Drp1 representing P259 identified by lalign. ‘:’ and ‘.’ correspond to complete identity and sequence similarity, respectively. **(c**) P259 (purple) is derived from the GTPase domain of Drp1. Surface and ribbon diagrams of Drp1 structure (PDB, 4BEJ) with the GTPase, bundle signaling element (BSE), and stalk domains highlighted. (**d**) A heatmap representing sequence conservation of human proteins that contain a region similar to P259. Sequence conservation corresponds to the number of residues similar/identical to P259 from each sequence. (**e**) Binding of 100 nM recombinant human Drp1, Mff, and Mfn2 to 50 nM surface-conjugated P259. The obtained graph is of the mean standardized response and ± s.e. as a function of time for n = 3 individual experiments, with 3 technical replicates each. (**f**) K_D_ values for each protein-peptide interaction at 100 nM protein concentration (n = 3, with 3 replicates) (**g**) Co-immunoprecipitation of recombinant Drp1 (200 ng) with each adaptor (200 ng) in the presence/absence of 1 µM P259 (a representative blot of two independent experiments). Represented Western blot is cropped from a full blot shown in the Expanded Western Blots section of the Supplementary Information. (**h**) Relative GTPase activity of recombinant Drp1 in the presence of 1 µM of peptide (n = 3–5). Data are mean ± s.e. from 3–5 independent experiments per condition.

Using a previously reported rational peptide design approach^15^, we identified a specific inhibitor of the Mff-Drp1 protein-protein interaction (Fig. 1a). In brief, for two proteins to have an induced intermolecular interaction (Fig. 1a, left), intramolecular interactions in one or both proteins must be dissociated to expose the intermolecular binding site (Fig. 1a, middle). We reasoned that the intermolecular binding and intramolecular masking sites may be homologous and therefore searched for a short homology sequence between the interacting proteins as a potential protein-protein interaction inhibitor (Fig. 1a, right). An lalign algorithm identified a ten-amino-acid homologous sequence between Drp1 and Mff (Fig. 1b). P259 was derived from the Drp1_22-31_ sequence. However, to increase the solubility of the peptide, glutamine_27_ was substituted for a glutamate, as found in the Mff sequence. This substitution was not predicted to affect function based on a PROVEAN score^16^, which computationally predicts a mutation’s severity (Fig. S1A). The P259 sequence is derived from the surface of Drp1′s GTPase domain (Fig. 1c). Interestingly, a previously identified inhibitor of the Drp1-Fis1 interaction, P110, was also derived from the GTPase domain of Drp1, but is from the opposite side^6^. The sequence corresponding to P259 is highly-conserved both in Drp1 and Mff (Fig. S1B), suggesting that it serves an important function in both proteins. Both Drp1 and Mff have seven isoforms and the identified homology sequence is present in 6/7 Drp1 isoforms and 7/7 Mff isoforms (Fig. S1C). Isoform 7 of Drp1, which lacks the P259 homologous region, cannot be stimulated by Mff^17^, suggesting that a sequence regulating the Mff-Drp1 interaction is missing from it. To search for potential off-target interactions of the peptide, a BLAST search of the human genome was performed to identify proteins that contain P259-like sequences and their relative conservation. Only Mff and Drp1 have highly-conserved P259-like sequences (Fig. 1d).

### P259 is a specific inhibitor of the Drp1-Mff interaction *in vitro*

To determine binding affinities of P259 to each protein partner, P259 was conjugated to a graphene surface with attached transistors that measure electrical conductance across it. A change in electrical resistance, corresponding to peptide-protein binding (Fig. 1e), provided evidence of interaction with a K_D_ of 12 nM and 24 nM for Drp1 and Mff, respectively (Fig. 1f). There was only negligible binding of P259 to Mfn2, a similarly large GTPase that participates in mitochondrial fusion (Fig. 1e). We confirmed the selectivity of P259 as an inhibitor of Drp1/Mff interaction; P259 abolished Mff binding to co-immunoprecipitated recombinant Drp1 without affecting the binding of any other receptor (Fig. 1g). Although P259 was derived from the GTPase domain of Drp1, it had no effect on the GTPase activity of recombinant Drp1 (Fig. 1h). These data indicate that P259 is a specific inhibitor of the Mff/Drp1 interaction.

### Inhibiting the Drp1-Mff interaction abolishes basal mitochondrial fission and causes severe mitochondrial dysfunction

**Figure 2:**
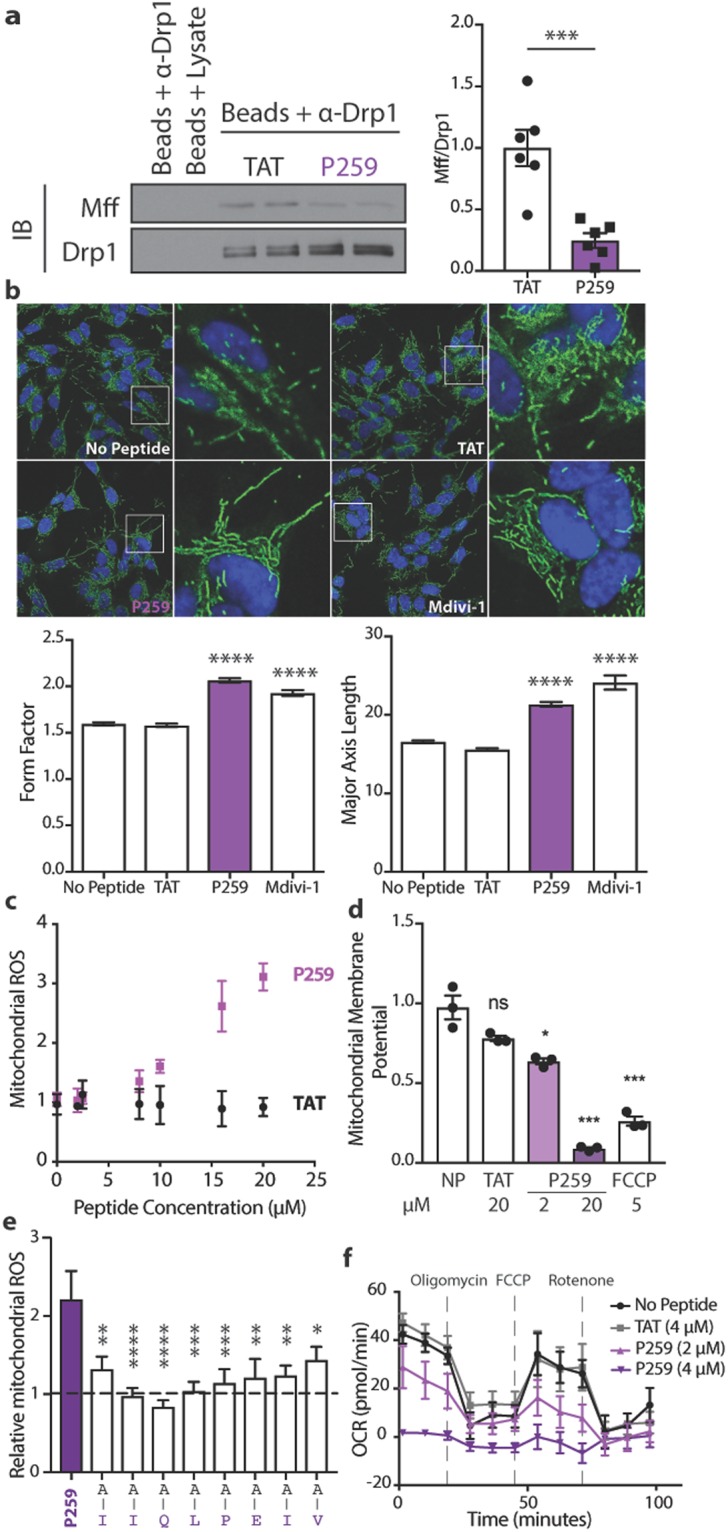
P259 inhibits the Mff/Drp1 protein-protein interaction and physiological mitochondrial fission in SH-SY5Y cells, resulting in mitochondrial dysfunction. (**a**) Co-immunoprecipiation of Drp1 and Mff from SH-SY5Y cell lysate treated with 1 µM P259 for 1 hour (n = 3, with 2 replicates). Represented Western blot is cropped from a full blot shown in the Expanded Western Blots section of the Supplementary Information. (**b**) Immunofluorescence images depicting mitochondrial morphology after a 30-minute treatment with 4 µM TAT, 4 µM TAT-P259 (P259), or 10 µM Mdivi-1. Hoechst 33342 is shown in blue and Tom20 in green (n = 3, 10 images each). Below, quantification of the form factor (degree of branching) and major axis length (length of mitochondria). At least 6,000 mitochondrial fragments were measured for each condition. Mitochondrial reactive oxygen species (ROS) production (MitoSox Red) (**c**) and mitochondrial membrane potential (TMRM) (**d**) after overnight treatment with indicated concentrations of peptide or FCCP. Data are from 3 independent experiments per condition normalized to the average no peptide value. (**e**) Relative mitochondrial ROS (MitoSox Red) signal in GFP positive cells expressing full-length or alanine-substituted P259 at indicated amino acids after two days of transfection. Values are normalized to a GFP empty vector (n = 3, with 3 replicates). (**f**) The effect of overnight treatment with 4 µM TAT or P259 on electron transport chain flux as measured through the oxygen consumption rate (OCR) (n = 3, with at least 3 replicates). Data are mean ± s.e.

We next sought to examine the effect of inhibiting the Drp1-Mff interaction on mitochondrial morphology and function in neuroblastoma cells. To enable P259 entry into cells, P259 was linked to a TAT_47–57_ carrier sequence through a glycine-serine-glycine linker (Fig. S1D). The TAT-carrier sequence facilitates peptide delivery through the cell membrane and the blood-brain barrier^7,18^. P259 treatment of neuroblastoma SH-SY5Y cells (1 µM) caused a significant reduction in Mff-Drp1 interactions relative to the TAT-carrier control treatment (Fig. 2a). A 30-minute P259 treatment resulted in more elongated and branched mitochondria, with increased major axis lengths and higher form factors, respectively (Fig. 2b). These effects were similar to treatment with Mdivi-1, a GTPase inhibitor of Drp1. Furthermore, P259 did not increase the rate of mitochondrial fusion, measured by photo-converted signal spreading of mitochondria-targeted photoconvertible Dendra2 protein (Fig. S2A). Therefore, this morphological phenotype is likely due to inhibition of mitochondrial fission, rather than activation of fusion, by inhibiting the Drp1-Mff interaction under basal conditions with P259.

Although excessive mitochondrial fission leads to mitochondrial dysfunction^5^, basal mitochondrial fission is necessary for the maintenance of mitochondrial function under physiological conditions. Indeed, inhibition of basal mitochondrial fission following an overnight treatment of P259 led to decline in mitochondrial integrity as measured by an increase in mitochondrial reactive oxygen species (ROS) levels (Fig. 2c), mitochondrial depolarization (Fig. 2d), as well as a dose-dependent decline in basal oxygen consumption rate and electron flux (Figs 2f, S2D). P259′s effect on mitochondrial ROS levels and membrane potential was dependent on Mff; Mff knockout MEFs were insensitive to P259 treatment in contrast to wild-type MEFs (Fig. S2E,F).

Using mitochondrial ROS as a phenotypic output, we determined which residues of P259 were necessary for its function. We transfected wild-type SH-SY5Y cells with a construct co-expressing GFP and the P259 peptide sequence alone (not including the TAT-carrier sequence; Fig. S2B). After a two-day expression, single-cell analysis of MitoSox signal in GFP-positive cells (Fig. S2C) demonstrated that in comparison to the empty GFP vector, expressing the P259 peptide doubled mitochondrial ROS levels (Fig. 2e). Mutation of each amino acid in the P259 sequence to an alanine demonstrated that each amino acid is required for its biological activity.

**Figure 3:**
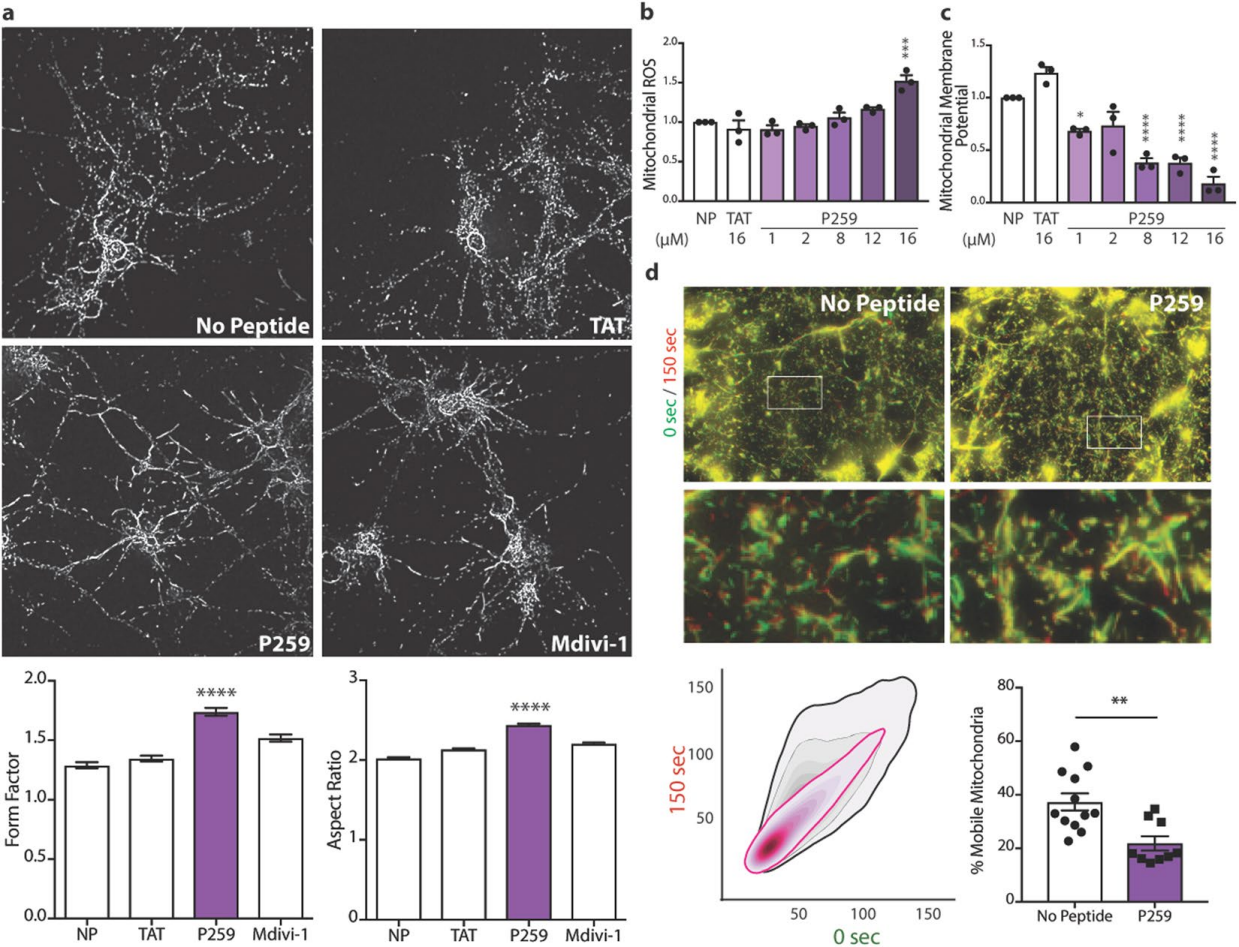
In primary cortical neurons, P259 inhibits basal physiological fission, resulting in mitochondrial dysfunction and loss in motility. (**a**) Top, immunofluorescence images depicting mitochondrial morphology (Tom20) of E17 primary cortical neurons after a 2-hour treatment with 1 µM TAT, 1 µM P259, or 10 µM Mdivi-1 (n = 3, 10 images each). Bottom, quantification of the form factor (degree of branching) and aspect ratio (major/minor axis length). At least 10,000 mitochondrial fragments were analyzed for each condition. Mitochondrial reactive oxygen species production (MitoSox Red) (**b**) and membrane potential (TMRM) (**c**) after overnight treatment with indicated concentrations of peptide (n = 3, with 3 replicates). (**d**) E17 primary cortical neurons treated with 4 µM P259 or no peptide control and stained with 50 nM TMRM. Overlay of live microscopy imaging at time 0 (green) and 150 (red) seconds. Bottom left: the corresponding bivariate kernel density estimate plots of the 0 sec versus 150 sec TMRM signal at each pixel for the represented images (n = 4 control and 6 P259, 3 movies each). An estimation of the % mobile mitochondria from the Pearson coefficient of each set of images. Data are mean ± s.e.

Similar to cell lines, a two-hour treatment of primary cortical neurons from E17 rats with P259 resulted in more elongated and branched mitochondria, suggesting that P259 inhibits mitochondrial fission under physiological conditions in primary cortical neurons as well (Fig. 3a). An overnight treatment with P259 resulted in diminished mitochondrial function as evidenced by increased mitochondrial ROS and decreased membrane potential (Fig. 3b,c). Therefore, specific inhibition of the Mff-Drp1 interaction by P259 led to inhibition of Mff-dependent physiological mitochondrial fission and loss of mitochondrial function.

### Mff-specific recruitment of Drp1 is necessary for mitochondrial motility

Microtubule-facilitated mitochondrial transport is essential in all cells, especially in neurons, where mitochondria must traverse along the axons to exert their function at synaptic connections. Drp1 is likely necessary for mitochondrial motility as shown by siRNA knockdown studies^19,20^. However, since Drp1 is primarily cytoplasmic, whether Drp1 must be on the outer mitochondrial membrane to enable mitochondrial transport and which mitochondrial adaptor is necessary for Drp1′s anchoring are yet to be identified.

To address these questions, primary cortical neurons cultured *in vitro* for 10 days were treated with P259 for one hour and then imaged for 20 minutes. To quantify mitochondrial motility, the TMRM images were colored green and red at times 0 and 150 seconds, respectively. Mitochondrial motility corresponded to decreased co-localization of the two colors (Fig. 3d). P259 treatment resulted in a tighter signal overlap, corresponding to diminished mitochondrial movement. Under basal conditions, approximately 37% of mitochondria were mobile, consistent with previously reported values^21^, whereas P259 treatment resulted in ~22% mobility, or a 40% decline in the proportion of mobile mitochondria. Therefore, Mff-mediated anchoring of Drp1 on the outer mitochondrial membrane was necessary for mitochondrial motility in primary cortical neurons.

### P259 inhibited brain mitochondrial fission, diminished mitochondrial function, and altered motor and cognitive functions

**Figure 4:**
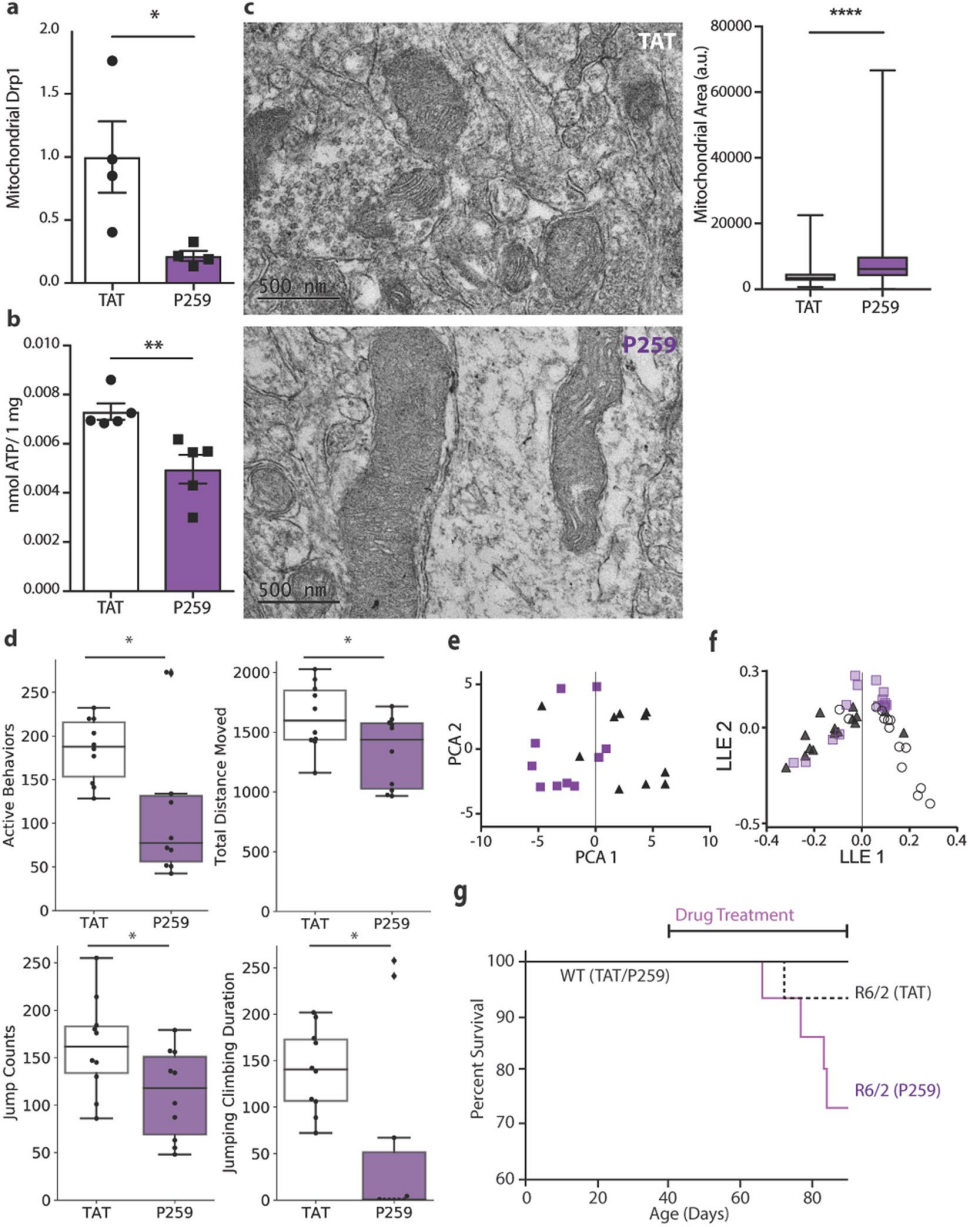
P259 inhibits physiological mitochondrial fission, lowers brain ATP levels and causes behavioral deficit in wild-type mice, and accelerates progression of disease in an R6/2 Huntington’s mouse model. WT and R6/2 mice were treated with 3 mg/Kg/day of P259 or TAT for 8 weeks starting at week 6 of age. (**a**) Quantification of mitochondrial Drp1 content relative to ALDH2 (a mitochondrial matrix enzyme) in the mitochondrial fraction isolated from total brain lysate of wild-type mice (Western blot shown in Fig. S3B) (n = 4 mice). (**b**) ATP content in brain tissue of wild-type mice (n = 4 mice, 2 replicates). (**c**) Representative transmission electron microscopy images of the striatum from wild-type mice. Quantification of mitochondrial area of 806 (TAT) and 1109 (P259) mitochondria from ten images per condition. (**d**) Active mouse behaviors (sum of time rearing, jumping and digging, in seconds), total distance moved (cm), jump counts, and combined jumping and climbing duration (sec) following 8 weeks of treatment with P259 (n = 10 mice). (**e**) Principal component analysis of the top 20% of behaviors collected from wild-type mice treated with 3 mg/Kg/day TAT (black)/P259 (purple) for 12 weeks. The first principal component represents 42% of the variation. (**f**) Local linear embedding dimensionality reduction and visualization of all behaviors collected weekly from TAT- (black) or P259- (purple) treated WT and TAT- (white) treated R6/2 mice over the course of 8 weeks. (**g**) Survival of WT and R6/2 mice treated with either TAT or P259 continuously at 3 mg/mL. All behavior and survival tests were conducted by an experienced observer who was blinded to the treatment groups. Data are mean ± s.e.

To determine the role of the Mff-Drp1 interaction *in vivo*, wild-type (WT) mice were treated with 3 mg/Kg/day of P259 using an Alzet osmotic pump implanted subcutaneously. We previously showed that this route effectively delivers TAT-conjugated peptides throughout the body, including across the blood-brain barrier^7,18^. Following an 8-week treatment, brain mitochondrial fraction was isolated and relative levels of Drp1 on the mitochondria were quantified. Treatment with P259 reduced mitochondrial Drp1 association by ~80% (Figs 4a, S3B), but had no effect on expression levels of both fission and fusion proteins as well as overall mitochondrial content and mitophagy (Fig. S3A,C). Whereas Drp1 has been previously shown to be necessary for mitophagy^22^, our results following prolonged inhibition of the Drp1/Mff interaction *in vivo* indicate that mitophagy may be dependent on an alternate Drp1 adaptor. P259 treatment led to larger and more elongated mitochondria in striatal neurons, shown by electron microscopy (Fig. 4c). The observed inhibition of basal fission in brains of wild-type mice was associated with a 30% decrease in ATP levels in the brain (Fig. 4b), suggesting a decline in mitochondrial function.

We also noted impairment in the behavior of the P259-treated wild-type mice as assessed by an activity chamber. After an 8-week treatment, P259-treated mice were less active, travelled for a shorter distance, and jumped and climbed less frequently (Fig. 4d). The first principal component of a two-dimensional principal component analysis (PCA) of the behaviors assessed successfully separated the behavior profile of P259- and TAT control-treated mice (Figs 4e, S4A). Of the behaviors tested weekly throughout treatment, jump counts/time, transition from the periphery to the center of the cage, movement counts, and stereotypic time (short movements) most greatly contributed to the difference between the TAT control-treated and the P259-treated mice (Fig. S4B). In all these behaviors, P259-treated wild-type mice performed worse than TAT-treated mice (Fig. S4A). Finally, local linear embedding (LLE) was used to reduce the dimensionality and visualize all behavior parameters collected weekly for 8 weeks for P259/TAT-treated WT mice and TAT-treated R6/2 Huntington’s disease (HD) mice (Fig. 4g). Huntington’s disease mice were used as a model of neurodegeneration associated with excessively fragmented mitochondria. In this comparison, WT mice treated with P259 appeared to have an intermediate aggregate behavior score between WT- and R6/2 control-treated mice. Cumulatively, these results suggest that P259 treatment leads to behavioral abnormalities in wild-type mice, suggesting that physiological fission through Mff is necessary for neurological function; inhibition of physiological fission alone through Mff is sufficient to cause neurological deficits.

### P259 diminished mitochondrial function in Huntington’s disease (HD) patient-derived fibroblasts and decreased survival in HD mice

We next determined the consequence of inhibiting Mff-mediated mitochondrial fission in Huntington’s Disease, which is characterized by excessive fragmentation of mitochondria. As we previously reported^23^, HD patient-derived fibroblasts have a ~40% higher mitochondrial ROS level relative to fibroblasts derived from healthy subjects (Fig. S2E) and treatment with P259 further increased mitochondrial ROS (Fig. S2G). Inhibition of mitochondrial fragmentation through the Fis1-Drp1 interaction with P110 has been previously shown to increase survival of HD R6/2 model mice^6^. Unlike P110, treatment with P259 led to a further decline in survival of the HD R6/2 model mice (Fig. 4h). These results suggest that even in an HD context, in which disease is associated with mitochondrial fragmentation, basal levels of fission through Mff are necessary. Inhibition of Mff-Drp1 interactions with P259 leads to accelerated disease progression, suggesting the importance of using an adaptor-specific inhibitor of pathological mitochondrial fission.

## Discussion

Excessive mitochondrial fission or fragmentation has been implicated in the pathogenesis of a variety of disorders, including cancer, as well as neurodegenerative and cardiovascular diseases^2,3^. It is therefore not surprising that targeting mitochondrial fission has been an attractive therapeutic approach. However, inhibiting mitochondrial fission can be a double-edged sword. Fission is essential for physiological mitochondrial functions, including mitochondrial quality control, intracellular distribution through motility, proper segregation during division, and interactions with other organelles^1,5^.

How can physiological mitochondrial fission be distinguished from physiological fission? Powerful genetic tools to abrogate expression of Drp1 or any of its adaptors have been immensely useful in implicating the role of each adaptor in facilitating mitochondrial fission. Recently, a collection of MEFs lacking mitochondrial fission adaptors showed that multiple receptors must be knocked out to achieve significant elongation of mitochondria^24^. This suggests that adaptors work cooperatively to facilitate Drp1-dependent mitochondrial fission, and/or that in a cell knockout model, adaptors may compensate for one another. To complement these studies, we developed pharmacological inhibitors to differentiate between pathological and physiological fission through specific Drp1-adaptor interactions.

Our study demonstrates that P259 is a useful pharmacological tool to study the physiological roles of Drp1 extending beyond fission. We were able to show that Mff-anchored Drp1 is necessary for maintaining mitochondrial motility in cortical neurons. This is consistent with previous work implicating Drp1 in enabling mitochondrial localization^19,20^, but now identifies Mff as a critical component in this process. Furthermore, Drp1 has been shown to anchor mitochondria to other subcellular structures, including the cytoskeletal network and endoplasmic reticulum^5^. P259 could be used as a tool to study whether Mff is specifically responsible for these inter-organelle tethering mechanisms.

A tremendous advance in the field has occurred with the development of Mdvi-1, a small-molecule inhibitor of the GTPase activity of Drp1^25^. However, Mdivi-1 cannot distinguish between Drp1′s physiological and pathological activities. We previously showed that P110, the Fis1 adaptor-specific inhibitor of Drp1, rescues pathological fragmentation but does not interfere with basal fission^6–8^. Here we showed that P259, the Drp1/Mff-specific inhibitor, hinders physiological fission and thus causes mitochondrial dysfunction. Strikingly, while inhibiting the interaction between Drp1 and Fis1 slowed the progression of neurodegenerative disorders^7–10^, treatment with the Drp1/Mff-specific inhibitor accelerated disease progression in a mouse model of Huntington’s Disease. These results indicate that even in disorders characterized by excessive mitochondrial fragmentation, physiological fission mediated through Mff is essential. Therefore, when considering targeting mitochondrial fragmentation as therapeutic approach, it is necessary to spare physiological Drp1 activity through inhibiting only adaptor-specific interactions.

## Methods

### Cell Lines, Primary Cells and Peptide Treatment

Human neuroblastoma SH-SY5Y cells were cultured in a 50/50 mixture of Dulbecco’s modified Eagle’s medium/Ham’s F-12, 10% FBS, and 1% penicillin-streptomycin solution. P259 treatment of SH-SY5Y cells was carried out in the same growth medium with the exception of using only 0.5% FBS.

Primary cortical neurons were isolated as previously described^26^. Cells were cultured for 10 days in serum-free NbActiv1 media prior to peptide treatment in the same media.

### Mitochondrial ROS and membrane potential

Following treatment, cells were washed with treatment media. Media was replaced with 100 µL staining solution: 50 nM TMRM or 5 µM MitoSox Red, 100 nM MitoTracker Deep Red, and 500 nM Hoechst in either Dulbecco’s modified Eagle’s medium or 50/50 mix of Dulbecco’s modified Eagle’s medium/Ham’s F-12, with 25 mM HEPES, and without phenol red. Cells were incubated in a 37 °C incubator for 30 minutes. After incubation with the staining solution, the staining solution was diluted 1:2.

Fluorescence images were automatically acquired using a BZ-X700 epifluorescence microscope with a 10X objective (Keyence). The Cy5 filter was used to autofocus on the mitochondria plane. Nuclei were imaged with a DAPI filter and the mitoROS (MitoSox) or membrane potential (TMRM) image was acquired with a TRITC filter. A MATLAB script was used to segment and count the number of nuclei and create a mitochondrial mask. MitoROS was calculated as the average MitoSox signal per cell. The mitochondrial membrane potential was calculated as the average TMRM signal in the mitochondrial mask.

### Peptide Treatment in Mice

Five-week old R6/2 HD mice and their wild-type littermates (a total of 60 mice) were implanted subcutaneously with a 28-d Alzet osmotic pump with either P259-TAT or TAT control peptide (15 mice per group) to be delivered continuously at a rate of 3 mg/Kg/day, as previously described^8^. All mice were implanted twice for a total of an 8-week treatment. 20 of the WT mice were implanted 3 times for a total of a 12-week treatment at 3 mg/Kg/day. All the experiments were in accordance with protocols approved by the Institutional Animal Care and Use Committee of Stanford University and were performed based on the National Institutes of Health Guide for the Care and Use of Laboratory Animals. All methods were carried out in accordance with the relevant guidelines and regulations.

### Statistical Analysis

Statistical analyses for all studies were processed using Prism software (version 7; GraphPad Software). Data are presented as mean ± s.e., and statistical significance was defined as P < 0.05 (*^,^**^,^ ***^,^****, correspond to p < 0.05, < 0.01, < 0.001, and 0.0001, respectively). Statistical significance was determined using an unpaired, two-tailed t test for results in Figs 1f,h, 2a,b,d, 3a–d, 4a–d, S2D and S3A. Significance in Fig. 2e was calculated using a Dunnett’s test compared to P259-GFP.

Detailed protocols are provided in the *Supplementary Methods* section. The datasets generated and analyzed during the current study are available from the corresponding author on reasonable request.

## Electronic supplementary material

Supplementary Information

## References

[1] Youle RJ, Van Der Bliek AM (2012). Mitochondrial fission, fusion, and stress. Science.

[2] Kornfeld OS (2015). Mitochondrial reactive oxygen species at the heart of the matter: new therapeutic approaches for cardiovascular diseases. Circulation Research.

[3] Archer SL (2013). Mitochondrial dynamics–mitochondrial fission and fusion in human diseases. The New England journal of medicine.

[4] Varga ZV, Ferdinandy P, Liaudet L, Pacher P (2015). Drug-induced mitochondrial dysfunction and cardiotoxicity. Am J Physiol Heart Circ Physiol.

[5] Joshi AU, Kornfeld OS, Mochly-Rosen D (2016). The entangled ER-mitochondrial axis as a potential therapeutic strategy in neurodegeneration: A tangled duo unchained. Cell Calcium.

[6] Qi X, Qvit N, Su Y-C, Mochly-Rosen D (2013). A novel Drp1 inhibitor diminishes aberrant mitochondrial fission and neurotoxicity. Journal of Cell Science.

[7] Guo X (2013). Inhibition of mitochondrial fragmentation diminishes Huntington disease – associated neurodegeneration. Journal of Clinical Investigation.

[8] Disatnik M-H (2016). Potential biomarkers to follow the progression and treatment response of Huntington’s disease. The Journal of Experimental Medicine.

[9] Joshi AU, Saw NL, Shamloo M, Mochly-Rosen D (2018). Drp1/Fis1 interaction mediates mitochondrial dysfunction, bioenergetic failure and cognitive decline in Alzheimer’s disease. Oncotarget.

[10] Joshi AU (2018). Inhibition of Drp1/Fis1 interaction slows progression of amyotrophic lateral sclerosis. EMBO Molecular Medicine.

[11] Prudent J (2015). MAPL SUMOylation of Drp1 Stabilizes an ER/Mitochondrial Platform Required for Cell Death. Molecular Cell.

[12] Calì T, Szabadkai G (2018). Organelles: the emerging signalling chart of mitochondrial dynamics. Current Biology.

[13] Loson OC, Song Z, Chen H, Chan DC (2013). Fis1, Mff, MiD49, and MiD51 mediate Drp1 recruitment in mitochondrial fission. Mol Biol Cell.

[14] Liu R, Chan DC (2015). The mitochondrial fission receptor Mff selectively recruits oligomerized Drp1. Mol Biol Cell.

[15] Souroujon MC, Mochly-rosen D (1998). Peptide modulators of protein–protein interactions in intracellular signaling. Nature Biotechnology.

[16] Choi Y, Chan AP (2015). PROVEAN web server: a tool to predict the functional effect of amino acid substitutions and indels. Bioinformatics.

[17] Macdonald PJ (2015). Distinct splice variants of dynamin-related protein 1 differentially utilize mitochondrial fission factor as an effector of cooperative GTPase activity. Journal of Biological Chemistry.

[18] Begley R, Liron T, Baryza J, Mochly-Rosen D (2004). Biodistribution of intracellularly acting peptides conjugated reversibly to Tat. Biochemical and biophysical research communications.

[19] Zhao J (2013). Mitochondrial dynamics regulates migration and invasion of breast cancer cells. Oncogene.

[20] Desai SP, Bhatia SN, Toner M, Irimia D (2013). Mitochondrial localization and the persistent migration of epithelial cancer cells. Biophysical journal.

[21] Wang X, Schwarz TL (2009). Imaging Axonal Transport of Mitochondria. Methods in enzymology.

[22] Twig G (2008). Fission and selective fusion govern mitochondrial segregation and elimination by autophagy. The EMBO Journal.

[23] Hwang S, Disatnik M-H, Mochly-Rosen D (2015). Impaired GAPDH-induced mitophagy contributes to the pathology of Huntington’s disease. EMBO Molecular Medicine.

[24] Osellame LD (2016). Cooperative and independent roles of the Drp1 adaptors Mff, MiD49 and MiD51 in mitochondrial fission. J Cell Sci.

[25] Cassidy-Stone A (2008). Chemical inhibition of the mitochondrial division dynamin reveals its role in Bax/Bak-dependent mitochondrial outer membrane permeabilization. Dev Cell.

[26] Shamloo M (2005). Death-associated Protein Kinase Is Activated by Dephosphorylation in Response to Cerebral Ischemia. Journal of Biological Chemistry.

