# Supplementary material for: Interaction of mitochondrial fission factor with dynamin related protein 1 governs physiological mitochondrial function in vivo
Source: Sci Rep. 2018 Sep 19;8:14034. doi: 10.1038/s41598-018-32228-1 (PMC6145916; doi:10.1038/s41598-018-32228-1)
Supplement: Supplementary file 1 — Supplementary Information [file 41598_2018_32228_MOESM1_ESM.pdf]

Interaction of mitochondrial fission factor with dynamin related protein 1 governs physiological mitochondrial function *in vivo*

Opher S. Kornfeld, Nir Qvit, Bereketeab Haileselassie, Mehrdad Shamloo, Paolo Bernardi, and Daria Mochly-Rosen

Supplementary Information

Supplementary Figures

Supplementary Figure 1. P259 represents a highly conserved homology region in the Drp1 (DNM1L) and MFF genes. a. The effect of an alanine (black) or glutamate (gray) substitution for each amino acid on the PROVEAN score for predicting mutation severity.[1] The data show that substitution of Q27 to E is unlikely to change the activity of the peptide. **b.** The sequence homology region corresponding to P259 (highlighted in purple) is highly conserved in both DNM1L and MFF throughout evolution. NCBI PSI-BLAST was performed for the human DNM1L (UniProt, O00429) and MFF (Uniprot, Q9GZY8) genes and the top 500 query hits were collected. Conservation score was defined as the number of sequences with exact residue identity corresponding to the human gene. **c.** The P259 homology region is conserved in 6/7 isoforms of DNM1L and 7/7 isoforms of MFF. **d.** P259 is composed of a $TAT_{47\text{-}57}$ carrier sequence linked to the Mff-Drp1 homology sequence by a Glycine-Serine-Glycine linker.

Supplementary Figure 2. P259 reduces mitochondrial function in cells. a. P259 does not affect mitochondrial fusion measured by photoconvertible dendra2 protein. Mito-dendra2 expressing MEFs were treated with 4 µM P259 for 15 minutes. Mitochondria images were captured using a GFP filter prior to photoconversion with DAPI filtered blue light. The photoconverted red signal was then imaged live for 5 minutes. The initial image following photoconversion was subtracted from each timed image to show change in signal over time. Each image series is representative of 9 movies collected over two independent experiments. **b.** Schematic diagram of the pSF-CMV-PGK-eGFP dual (independent) promoter GFP plasmid with P259 or its alanine substitutes expressed under the CMV promoter. **c.** Representative images showing GFP, related to P259 expression, and the corresponding mitochondrial reactive oxygen species production (MitoSox). **d.** P259 treatment reduces basal oxygen consumption of SH-SY5Y cells. Basal oxygen consumption rate reading of SH-SY5Y cells treated with indicated concentrations of P259 or with 4 µM TAT overnight. Data are of 3 independent experiments with the whiskers calculated by the Turkey method. **e.** P259 increases mitochondrial ROS production in an Mff-dependent manner. Mitochondrial reactive oxygen species production (measured by MitoSox) of WT and MFF knockout MEFs cells treated with 4 µM P259 daily for three days. MitoSox values were normalized to the average no peptide treatment in each cell line. Data are the mean ± s.e. from 3 independent experiments, with three technical replicates each (ns and *** correspond to $p = 0.23$ and <0.001, respectively, as determined by an unpaired, two-tailed t test). **f.** P259 decreases mitochondrial membrane potential in an Mff-dependent manner. Mitochondrial membrane potential (measured by TMRM) of WT and MFF knockout MEFs cells treated with 20 µM P259 overnight. TMRM values were normalized to the average no peptide treatment in each cell line. Data are the mean ± s.e. from 3 independent experiments, with three technical replicates each (ns and ** correspond to $p = 0.93$ and <0.01, respectively, as determined by an unpaired, two-tailed t test). **g.** Treatment with P259 further increased mitochondrial ROS production in fibroblasts derived from a Huntington's disease patient. Mitochondrial ROS (MitoSox) from fibroblasts derived from a Huntington's disease patient were treated with the indicated peptide concentration daily for three days. Mitochondrial ROS values were normalized to the average MitoSox signal from the average healthy patient fibroblast control (no peptide treatment). Data are mean ± s.e. from 3 independent experiments.

Supplementary Figure 3. P259 reduces accumulation of Drp1 on the outer mitochondrial membrane in brains of wild-type mice, while not affecting expression levels of other fission and fusion machinery proteins. Total lysate (**a,c**) and mitochondrial fraction (**b**) from brain tissue dissected from mice treated with 3 mg/Kg/day P259 or TAT control for 8 weeks. Represented western blots are cropped from full blots shown in the Expanded Western Blots section of the Supplementary Information. Represented western blots are cropped from a full blots shown in the Expanded Western Blots section of the Supplementary Information.

Supplementary Figure 4. Principal component analysis of behaviors from wild-type mice treated with P259. a. The top 20% of behaviors collected from wild-type mice treated with 3 mg/Kg/day TAT (blank) or P259 (purple) for 12 weeks, as determined by a combination of the ANOVA F- and mutual information scores for each behavior (week designation corresponds to mouse age when the behavior was observed). **b.** The eigenvector of the first principal component, representing 42% of the variation of the data.

Supplementary Figure 1

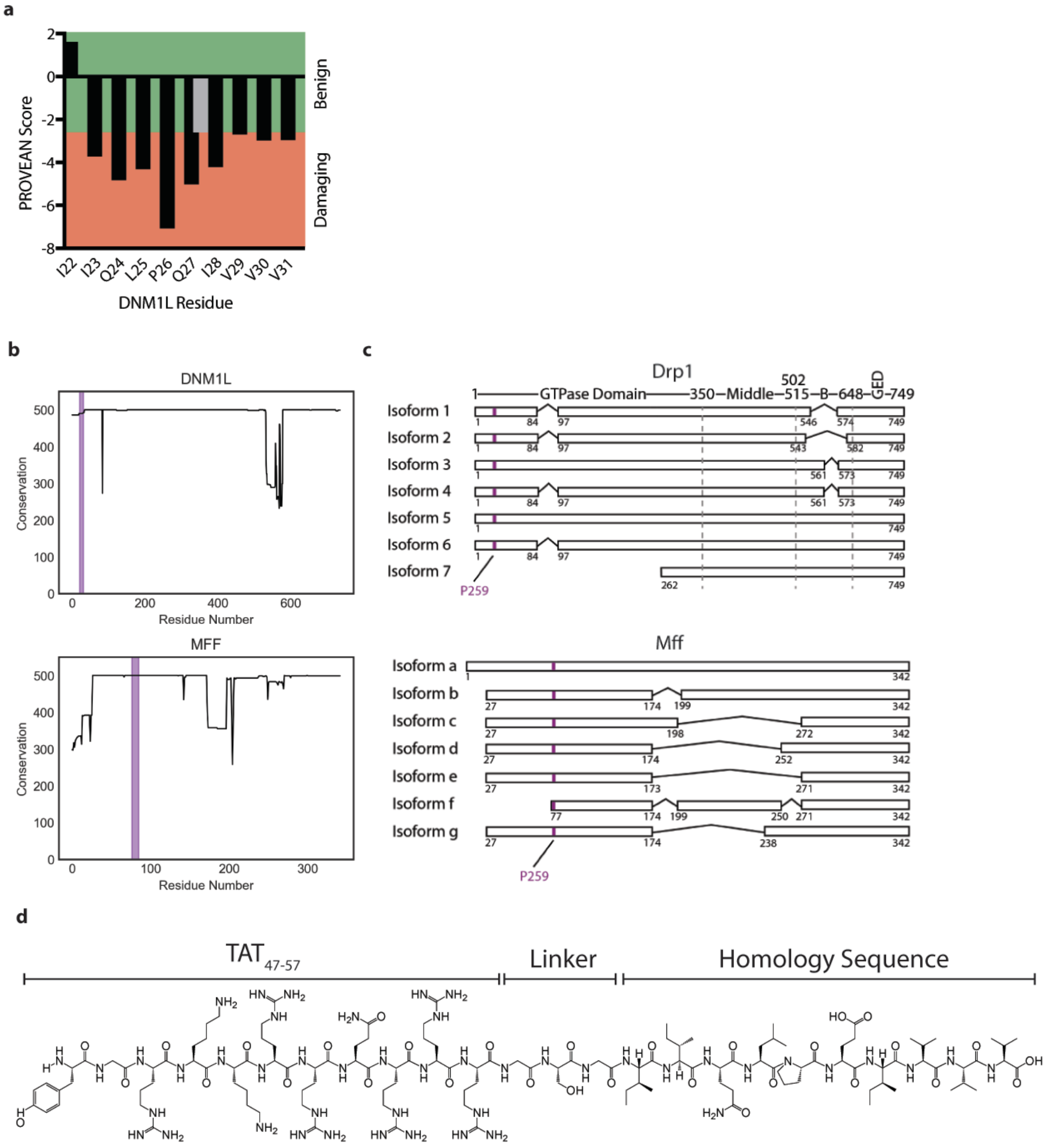

Supplementary Figure 2

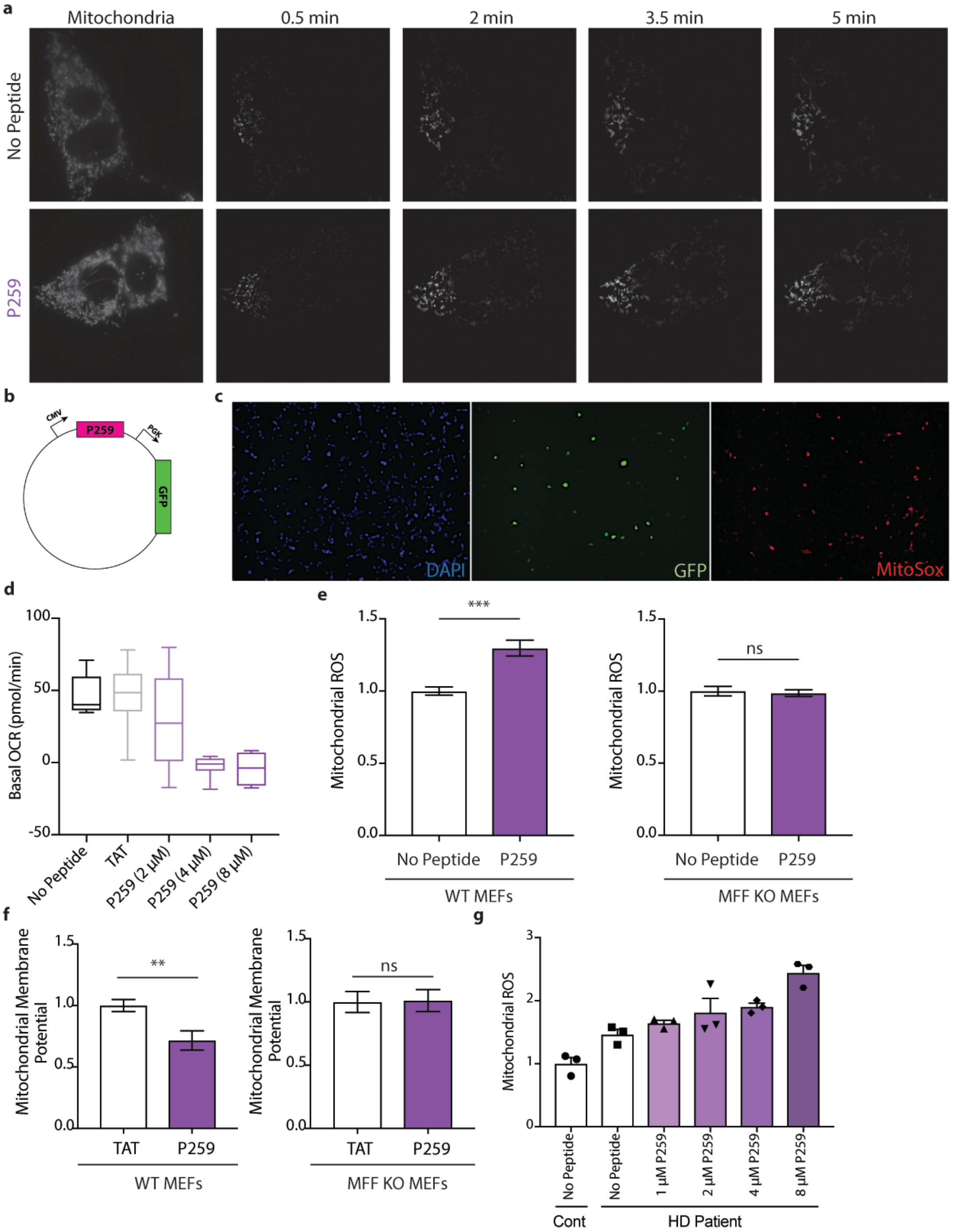

Supplementary Figure 3

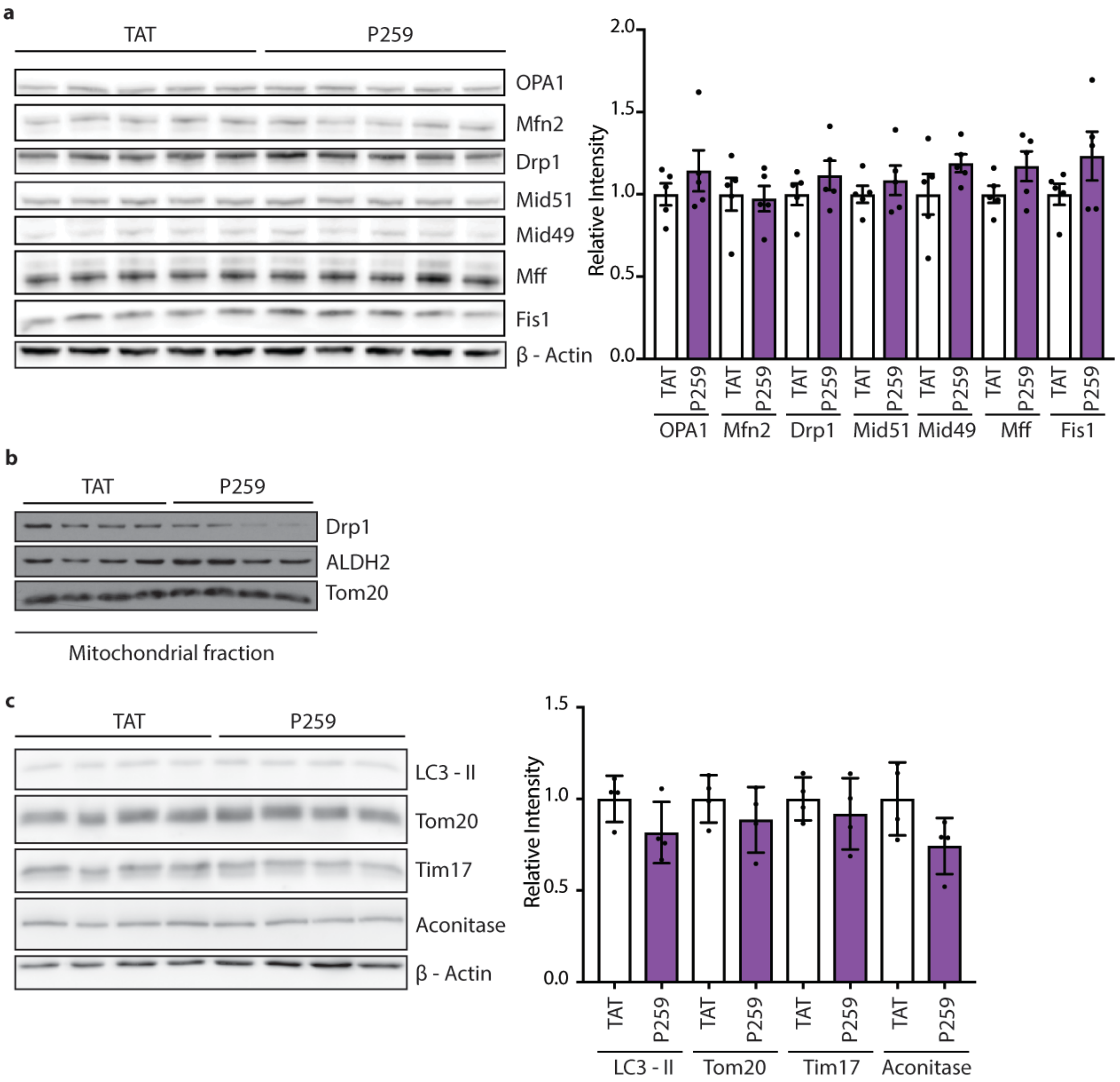

Supplementary Figure 4

a

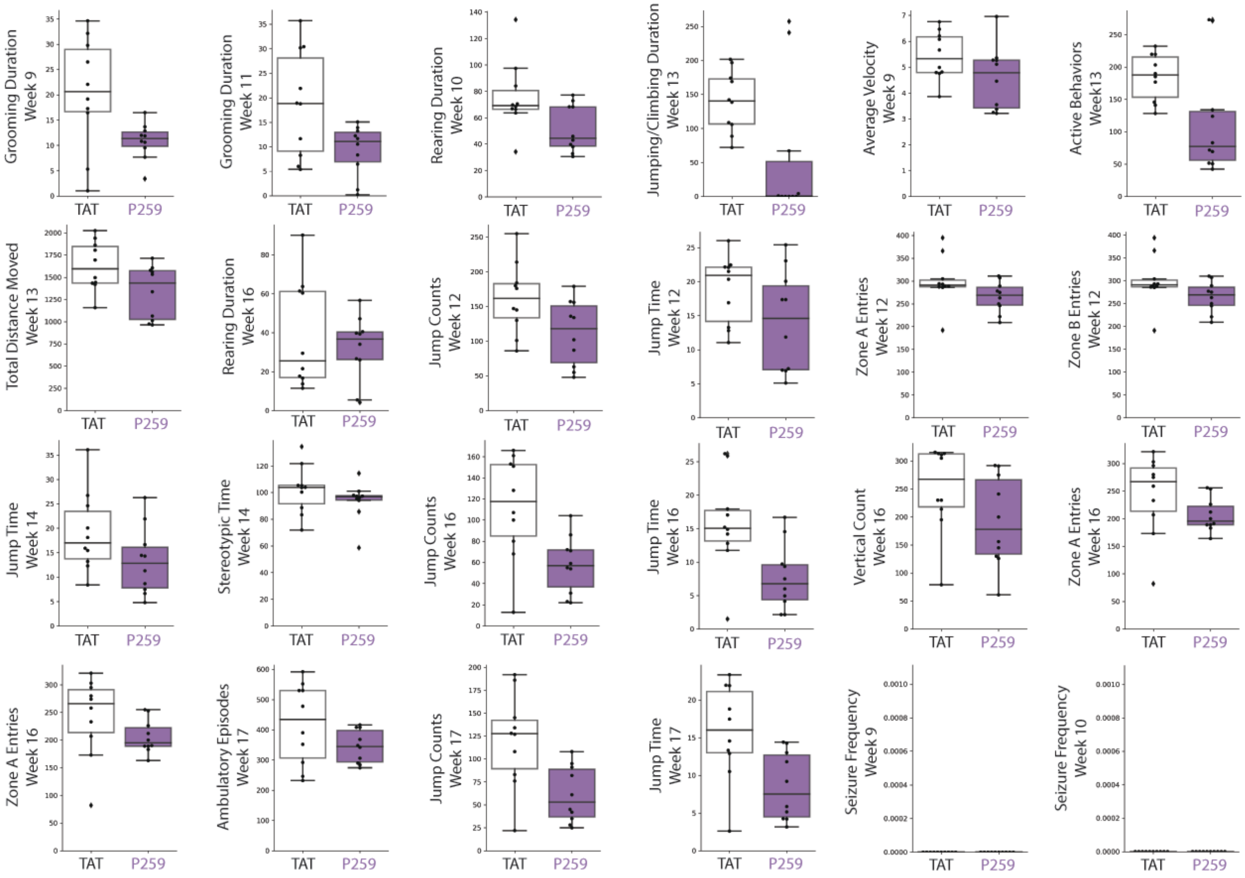

b

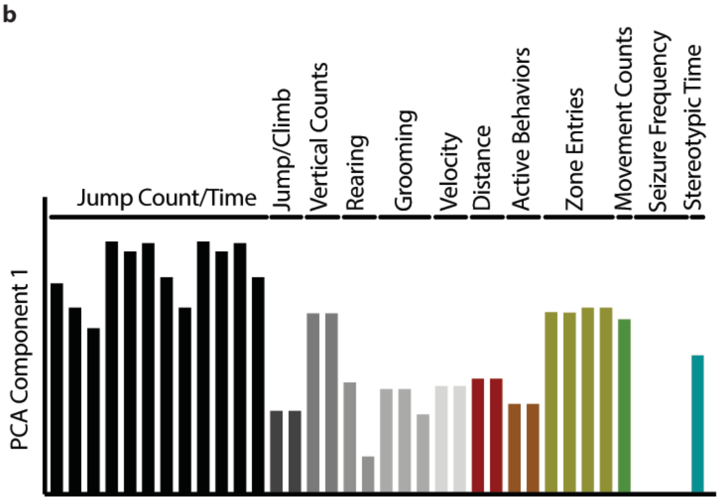

Expanded Western Blots:

Dashed line (- -) represents membrane strips incubated with the indicated antibody. Solid line (-) represents cropped area included in the indicated figure.

Figure 1g

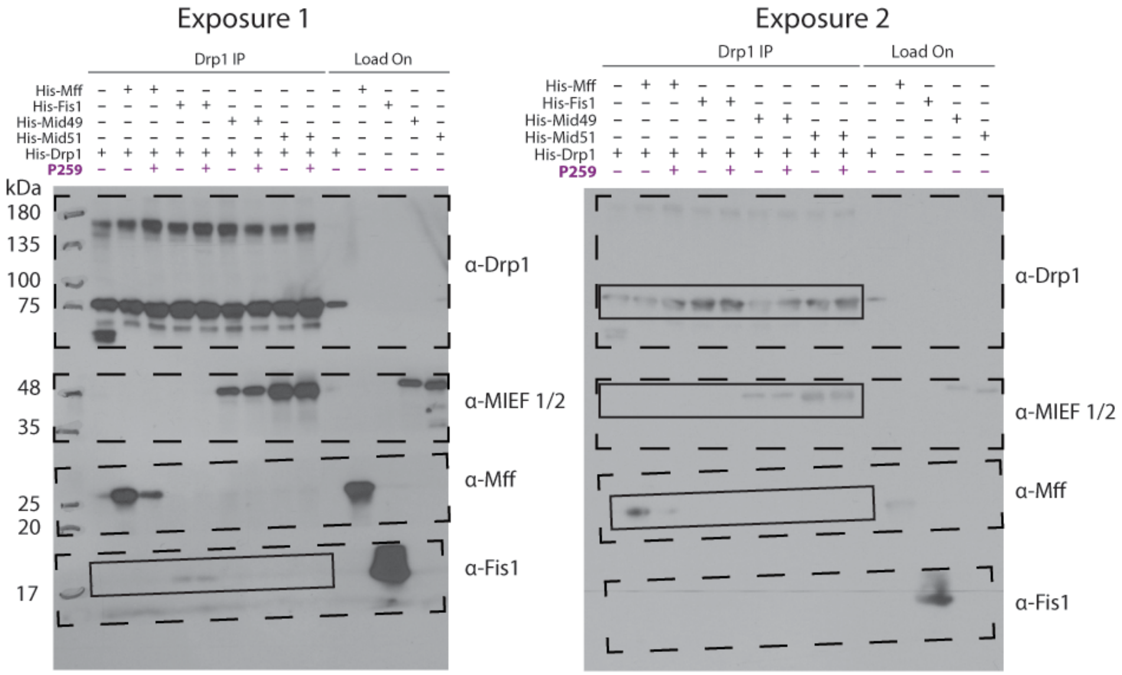

Figure 2a

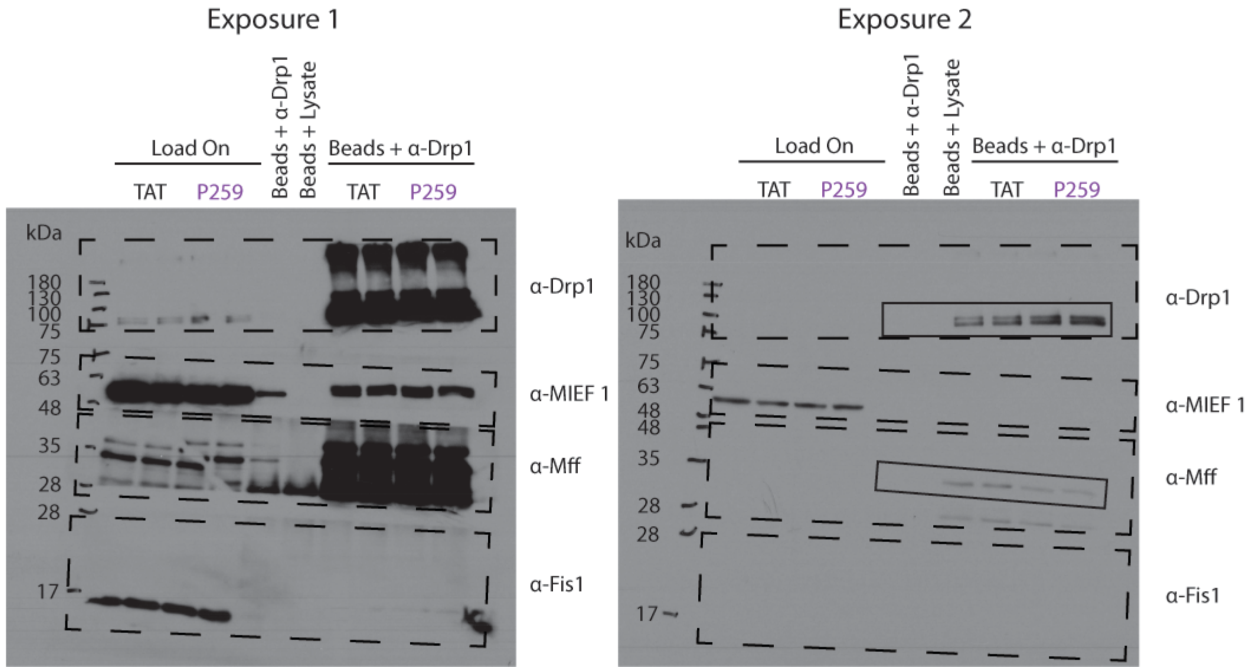

Supplementary Figure 3a

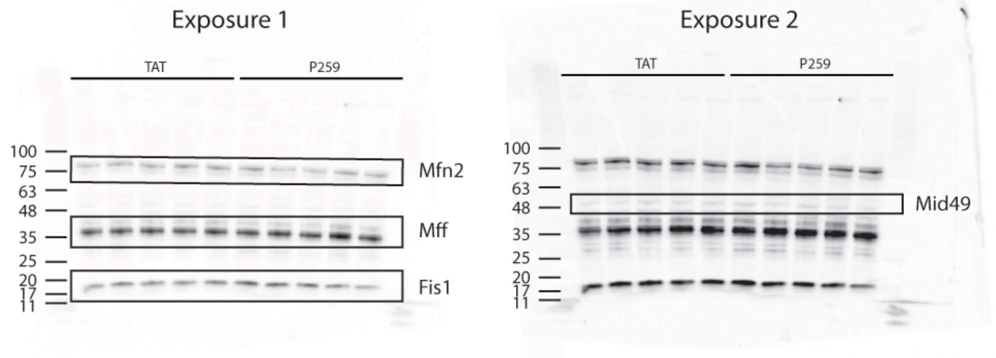

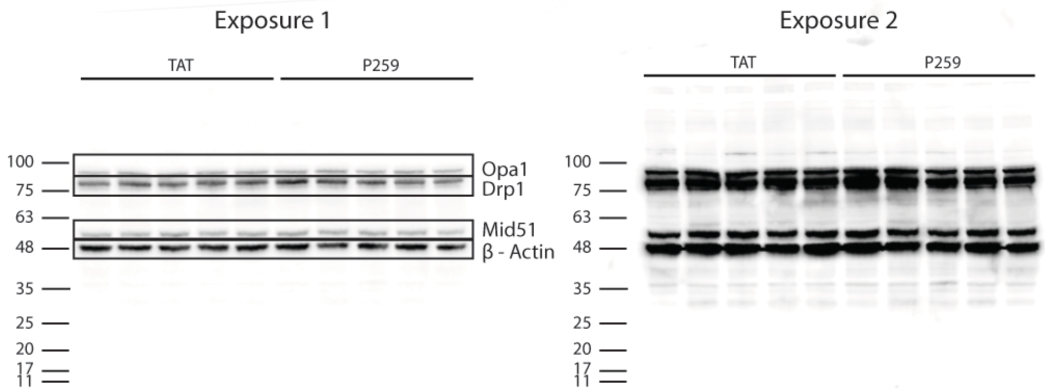

Supplementary Figure 3b

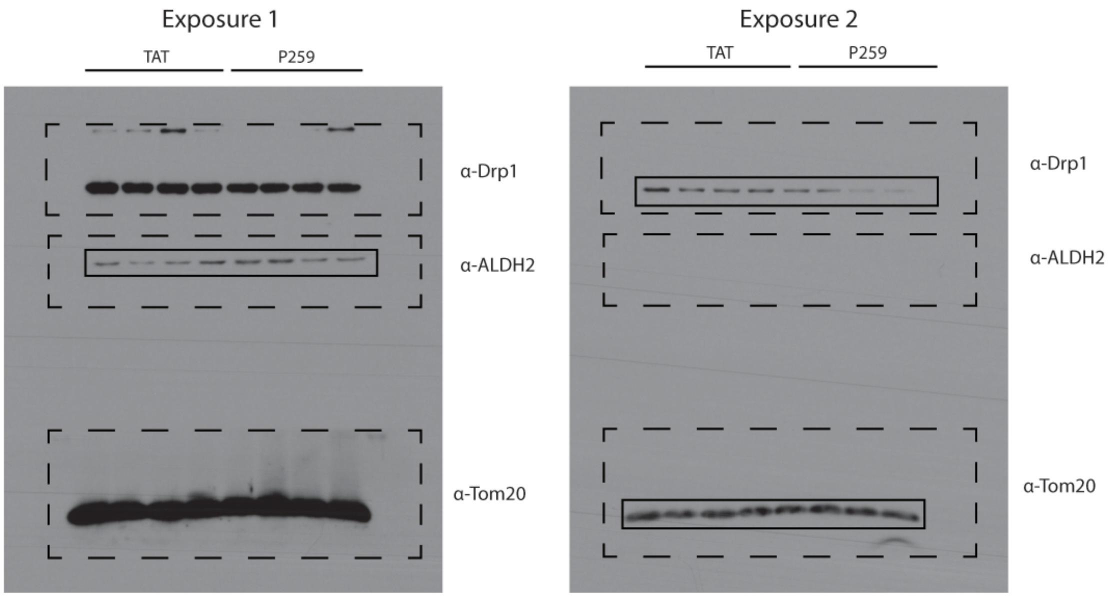

Supplementary Figure 3c

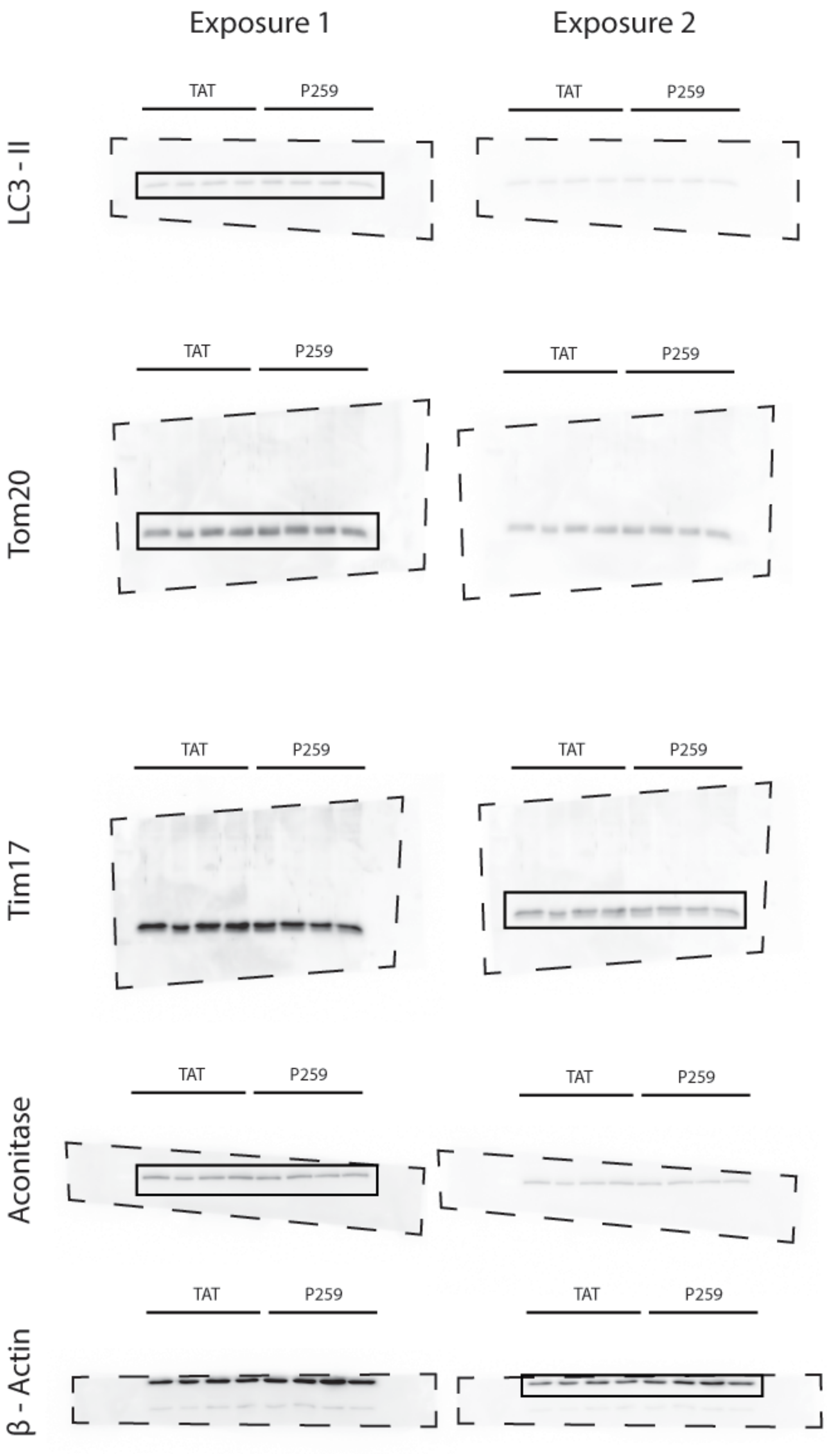

Supplementary Methods:

Cell lines

Human neuroblastoma SH-SY5Y cells purchased from ATCC were cultured in a 50/50 mixture of Dulbecco's modified Eagle's medium/Ham's F-12 (Corning, NY, USA), 10% Fetal Bovine Serum (FBS) (Gemini, CA, USA), and 1% penicillin-streptomycin solution (Corning, NY, USA). Assays using SH-SY5Y cells were carried out in the same growth medium with the exception of using only 0.5% FBS.

Wild-type and MFF knockout mouse embryonic fibroblast (MEF) cells were generously provided by Professor David Chan. MEF cells were cultured in Dulbecco's modified Eagle's medium (Corning, NY, USA), 10% Fetal Bovine Serum (FBS) (Gemini, CA, USA), and 1% penicillin-streptomycin solution. Assays using MEF cells were carried out in the same growth medium supplement with only 0.5% FBS.

HD 33-year-old male patient-derived fibroblast cells line (GM04693, Coriell Institute, NJ, USA) and control patient (HDFa, Thermo Fisher, MA, USA) were cultured in minimum essential media (Corning, NY, USA), 15% Fetal Bovine Serum (FBS) (Gemini, CA, USA), and 1% penicillin-streptomycin solution (Corning, NY, USA), as we previously described.[2] Assays using fibroblasts were carried out in the same growth medium without any added FBS.

Primary rat cortical neurons

Primary cortical neurons were isolated as previously described. Briefly, cortical tissue was dissected from E17 Sprague Dawley rats (Charles River Laboratories) and dissociated with papain (BrainBits, IL, USA) followed by manual pipetting. The resulting supernatant was centrifuged at 1100 rpm for 1 min, and cell pellet was resuspended in serum-free NbActiv1 media (BrainBits, IL, USA). Cells were cultured for 10 days prior to peptide treatment.[3]

Rational peptide design

A rational peptide design approach, as previously published, has been followed to find a specific inhibitor of the Drp1-Mff interaction [4]. In summary, human DNM1L (Drp1) (O00429; UniProtKB) and MFF (Q9GZY8; UniProt KB) protein sequences were aligned by Pearson's lalign program on the ExPASy server. All default parameters were utilized with the following exceptions: scoring matrix (BLOSUM80), opening gap penalty (-14), and extending gap penalty (-4).[5]

To determine how conserved the resulting alignment region is, a PSI-BLAST was performed on both the human DNM1L and MFF sequences and the top 500 query hits were obtained.[6] The homology region was identified in each multiple sequence alignment and conservation score was defined as the number of sequences with an exact match at each residue of the human DNM1L and MFF proteins. The alignment across isoforms was obtained using the DNM1L (NP_036192.2, NP_036193.2, NP_005681.2, NP_001265392.1, NP_001265393.1, NP_001265394.1, NP_001265395.1; NCBI) and MFF (NP_001263990.1, NP_001263991.1, NP_001263992.1, NP_001263993.1, NP_001263994.1, NP_001263996.1, NP_001263997.1; NCBI).

To obtain the heat-map of proteins that have sequences homologous to the P259 sequence, an NCBI protein blast of the P259 sequence (IIQLPEIVVV) was performed across the human genome database and the top 75 gene query hits were recorded.[7] The orthologs of each available gene were obtained from the Ensembl database.[8] Of the resulting 75 human gene hits, 37 had orthologs in the Ensembl database from 31 species that have the P259 homology region in both

Drp1 and Mff. Utilizing BioPython, a pairwise alignment was performed between each ortholog and the P259 sequence, with a modified alignment scoring matrix (1 for similar/identical residue, 0 otherwise).

Peptide synthesis

P259 was synthesized using the same protocol as previously published by the lab (Qi, 2013). In brief, an automated microwave peptide synthesizer (Liberty, CEM Corporation) was used to perform solid phase peptide synthesis by a series of coupling of fluorenylmethoxycarbonyl (Fmoc)-protected amino acids and hydroxybenzotriazol (HOBt) deprotection reaction steps. Peptide identity and purity was confirmed by matrix-assisted laser desorption/ionization mass spectrometry and reverse-phase high-pressure liquid chromatography (Shimadzu, MD, USA), respectively. TAT_{47-57} control peptide was purchased from Bachem (4048761, Switzerland).

Recombinant protein synthesis

Bacterial expression of Drp1 (DNM1L) and the soluble regions of Mff (1-198), Fis1 (1-119), Mid49 (48-454), and Mid51 (47-463) was performed using cDNA obtained through a generous gift by Professor Janet Shaw. The cDNA of each protein obtained from yeast colonies was inserted into the pET-28a vector with a 6xHis tag (EMD Millipore, Darmstadt, Germany) by Gibson cloning (E2611S, NEB, MA, USA). Proteins were expressed in BL21 *E. coli*. pET-28a vector-containing bacteria were grown at 37 °C until OD_{600} = 0.7. Protein expression was induced with 0.1 mM IPTG overnight at 37 °C. Bacteria were then lysed by sonication in lysis buffer [50 mM Tris, 300 mM NaCl, 5% glycerol, 0.4 mM PMSF, 1 mg/mL lysozyme, 0.1% Triton X-100, and protease inhibitor cocktail (Sigma, MO, USA) at pH 7.8]. After centrifugation at 30,000g for 30 minutes, the supernatant was loaded onto Ni-columns (HisTrap columns, GE Healthcare, PA, USA) for 1 hour at 4 °C while shaking. The beads were washed twice with wash buffer (20 mM sodium phosphate, 0.5 M NaCl, 20 mM imidazole, pH 7.4) and eluted in elution buffer (20 mM sodium phosphate, 0.5 M NaCl, 500 mM imidazole, pH 7.4). The eluted fractions were concentrated using Vivaspin-6 MWCO spin columns (GE Healthcare) and stored in 40% glycerol to 60% elution buffer. Mfn2 recombinant protein was purchased from Origene (TP326143, MD, USA).

Peptide binding to recombinant proteins

An AGILE-R100 sensor device and the corresponding COOH sensor chips (Nanomedical Diagnostics Inc, CA, USA) were used to determine relative recombinant protein affinities to an immobilized peptide. The P259-TAT peptide was conjugated to the graphene surface of the sensor chips using N-Ethyl-N'-(3-dimethylaminoproply)carbodiimide hydrochloride (EDC)/N-Hydroxysulfosuccinimide (sNHS) coupling chemistry, as described by the manufacturer. Briefly, the graphene surface of the chips was washed with 30 μL of 50 mM 2-(N-morpholino)ethanesulfonic acid (MES) buffer, pH 6.0 (E183, Amresco). The chip surface was activated by a 15-minute incubation with 2 mM EDC (CAS No. 25952-53-8) and 5.5 mM sNHS (CAS No. 106627-54-7) in 50 mM MES buffer at pH 6.0. The activated surface was then incubated with 50 mM P259 in 50 mM MES pH 6.0 for 30 minutes. Coupling was quenched and the surface was blocked as instructed by the manufacturer. The chip surface was then washed with RIPA buffer (150 mM NaCl, 1.0% Triton X-100, 0.5% deoxycholate, 0.5% sodium dodecyl sulfate, 50 mM Tris, pH 8.0).

Protein binding to the surface was obtained through current measurements during a voltage sweep by a potentiostat. Binding of a protein to the peptide at the interface between the surface and a liquid droplet changes the resistance of the sensor. This change in resistance is proportional to the number of protein molecules bound to the conjugated peptide. Three separate transistors were utilized as technical replicates to measure the sensor response. After calibration with 30 μL RIPA buffer for 3 minutes, 30 μM of 100 nM protein was incubated over the surface for 3 minutes as sensor response was measured. Dissociation was measured by re-applying 30 μL of RIPA buffer. Each measurement was repeated three times, with three technical replicates each.
The binding curve for each measurement was fitted to the following equation:

$$s(t) = (s(0) - \text{Plateau})e^{-k_{obs} \cdot t} + \text{Plateau}$$

Where *s* is the sensor response as a function of time (*t*). The plateau corresponds to the sensor response at equilibrium and k_{obs} is defined as the following:

$$k_{obs} = k_{on}[A] + k_{off}$$

The standardized response value was calculated by the following equation:

$$1 - \frac{s(t) - \text{Plateau}}{s(0) - \text{Plateau}}$$

K_D values were subsequently calculated using the binding and dissociation curves and the analysis software provided by the manufacturer (Nanomedical Diagnostics).

Co-immunoprecipitation

Co-immunoprecipitation of recombinant protein: 20 μL of Dynabeads Protein G (Thermo Fisher, MA, USA) per sample were incubated with 1 μg anti-Drp1 mouse antibody (611113, BD Biosciences, CA, USA) for 30 minutes at room temperature. Beads were then washed twice with 500 μL conjugation buffer (20 mM Sodium Phosphoate, 0.15 M NaCl, pH 7.5). Beads were resuspended in 500 μL of 5 mM BS3 (Thermo Fisher, MA, USA) in conjugation buffer and incubated for 30 minutes at room temperature. Conjugation was quenched by adding 25 μL of 1 M Tris pH 7.5. Beads were then washed twice with 500 μL RIPA buffer. Following conjugation, beads were incubated with 200 ng of Drp1 recombinant protein in 500 μL of RIPA buffer at 4 °C for 2 hours. Following Drp1 incubation, samples were washed 3 times with RIPA buffer and incubated with 200 ng of Mff/Fis1/Mid49/Mid51 either in the presence or absence of 1 μM P259 for 2 hours at 4 °C. Beads were finally washed three times with RIPA buffer. Beads were finally resuspended in 20 μL of Laemmli buffer.

Co-immunoprecipitation from cell lysate: Antibody conjugation to beads was carried out as described above. SH-SY5Y cell lysate was prepared as previously described.[9] Briefly, treated SH-SY5Y cells were washed with cold PBS prior to a 20-minute incubation in 1% formaldehyde. The formaldehyde crosslinking reaction was quenched by a 10-minute incubation in 100 mM glycine in PBS. Cells were scraped and lysed in 500 μL RIPA buffer using a 27.5-gauge needle. Protein content was measured using Bradford protein assay dye (Bio-Rad, CA, USA) and subsequently standardized. Cell lysate was incubated with beads overnight at 4 °C. Beads were

finally washed three times with RIPA buffer. Finally, beads were resuspended in 20 μL of Laemmli buffer.

Western blot analysis
Protein samples resuspended in Laemmli buffer were loaded onto SDS-PAGE and subsequently transferred to nitrocellulose membranes. The following primary antibodies were used in the study: Drp1 (BD Biosciences, 611113), Fis1 (Proteintech, 10956-1-AP), Mff (Proteintech, 17090-1-AP), Mfn2 (Proteintech, 12186-1-AP), Mid49 (Proteintech, 16413-1-AP), Mid51 (Proteintech, 20164-1-AP), Tom20 (Santa Cruze Biotech, sc-11415), VDAC1 (Abcam, 14734), β-actin (Cell Signaling Technology, 3700), OPA1 (BD Biosciences, 612607), 6xHis (Aviva, OAEA00010).

GTPase activity
The effect of peptides on GTPase activity of Drp1 was measured by incubating recombinant Drp1 (25 ng) with 1 μM peptide (TAT, P259, P110) for 15 minutes prior to the addition of 0.5 mM GTP for 1 hour at 37 °C. GTPase activity was measured using a colorimetric GTPase assay kit (Novus Biologicals, CO, USA) as instructed by the manufacturer.

Immunofluorescence
Cells were fixed onto Permanox plastic slides with 4% paraformaldehyde in PBS for 10 minutes at room temperature. Cells were permeabilized in 0.1% Triton X-100 and 1% normal goat serum (blocking buffer) for one hour at room temperature. Permeabilized cells were incubated overnight with primary anti-Tom20 antibody (Santa Cruze Biotech, sc-11415, 1:250 dilution) in a humidified chamber. Slides were subsequently incubated with FITC-conjugated goat anti-rabbit IgG for two hours at room temperature in a light-shielding humidified chamber. Cells were washed with blocking buffer and stained with Hoechst 33342 (Invitrogen, OR, USA, 1:10,0000). Coverslips were mounted with a Slowfade antifade solution (Invitrogen, OR, USA). Images were acquired using an LSM 700 confocal laser-scanning microscope equipped with a Plan Apochromat 63x/1.4 NA oil-immersion objective (Zeiss).

Mitochondrial morphology analysis
High-throughput image analysis of mitochondrial morphology was performed using MATLAB with the Image Processing toolkit. Background illumination, obtained by morphological opening of the image, was subtracted from the original image. The image was then passed through a top-hat transformation to highlight objects from the background followed by a pixel-wise adaptive low-pass Wiener filter to remove noise. A 90th percentile threshold was used to convert the image to a binary image. Erroneously large and small objects were removed from the image and morphological features were extracted from the binary objects. Major axis length is defined as the length of the longest line that can be drawn through a binary object while the minor axis length is defined as the length of the shortest line. The aspect ratio is defined by the major axis length divided by the minor axis length. Finally, the form factor (FF), a measure of the degree of branching, is defined as:

$$\mathrm{FF} = \frac{\mathrm{P}^2}{4\pi\mathrm{A}}$$

Where P is the perimeter of the object and A is the area.

Mitochondrial reactive oxygen species determination

SH-SY5Y neuroblastoma cells were treated overnight in a 96-well plate with increasing concentrations of P259 and TAT control peptides in a 50/50 mix of Dulbecco's modified Eagle's medium/Ham's F-12 (Corning, NY, USA), 0.5% Fetal Bovine Serum (FBS) (Gemini, CA, USA), and 1% penicillin-streptomycin solution (Corning, NY, USA). Primary cortical neurons were treated with increasing concentrations of peptides in NbActiv1 media. HD Patient- and adult-derived fibroblasts were treated once daily for three days with increasing concentrations of peptides in minimal essential medium (Corning, NY, USA) and 1% penicillin-streptomycin solution (Corning, NY, USA). MEFs were treated once daily for three days with 4 µM of P259 and TAT control peptides in Dulbecco's modified Eagle's medium (Corning, NY, USA), 0.5% Fetal Bovine Serum (FBS) (Gemini, CA, USA), and 1% penicillin-streptomycin solution (Corning, NY, USA).

After treatment, cells were washed with assay media, and the media was replaced with 100 µL staining solution: 5 µM MitoSox Red (Thermo Fisher, MA, USA), 100 nM MitoTracker Deep Red, and 500 nM Hoechst (Thermo Fisher, MA, USA) in either Dulbecco's modified Eagle's medium or 50/50 mix of Dulbecco's modified Eagle's medium/Ham's F-12, with 25 mM HEPES, and without phenol red. Cells were incubated in a 37 °C incubator for 30 minutes. After incubation with the staining solution, the staining solution was diluted 1:2 with either Dulbecco's modified Eagle's medium or 50/50 mix of Dulbecco's modified Eagle's medium/Ham's F-12, with 25 mM HEPES, and without phenol red.

Fluorescence images of the 96 well plate were automatically acquired using a BZ-X700 epifluorescence microscope with a 10X objective (Keyence). The Cy5 filter (Keyence) was used to autofocus on the mitochondria plane. Nuclei were imaged with a DAPI filter (Keyence) and the MitoSox mitochondrial ROS image was acquired with a TRITC filter (Keyence). A MATLAB script was used to segment and count the number of nuclei in each image. Mitochondrial ROS was calculated as the average MitoSox signal per cell.

Mitochondrial membrane potential determination

Cells were treated in 96-well plates as described above. After treatment, cells were washed with assay media. Media was replaced with 100 µL staining solution: 50 nM TMRM (Thermo Fisher, MA, USA), 100 nM MitoTracker Deep Red, and 500 nM Hoechst (Thermo Fisher, MA, USA) in either Dulbecco's modified Eagle's medium or 50/50 mix of Dulbecco's modified Eagle's medium/Ham's F-12, with 25 mM HEPES, and without phenol red. Cells were incubated in a 37 °C incubator for 30 minutes. After incubation with the staining solution, the staining solution was diluted 1:2 with either Dulbecco's modified Eagle's medium or 50/50 mix of Dulbecco's modified Eagle's medium/Ham's F-12, with 25 mM HEPES, and without phenol red.

Fluorescence images were captured as described above, with the exception of the TMRM mitochondrial membrane potential image being acquired with a TRITC filter (Keyence). Using MATLAB, the MitoTracker image converted to a binary mask of mitochondrial objects. The mitochondrial membrane potential was calculated as the average TMRM signal in the mitochondrial mask.

Live mitochondrial fusion assay

Wild-type MEF cells were seeded in in an 8-well glass chamber slide (ibidi, Martinsried, Germany) at 10,000 cells/well. Cells were transfected with 100 ng of the mito-dendra2 vector (Addgene, 55796, Professor David Chan) using 100 ng of lipofectamine 2000 (ThermoFisher, MA,

USA). Following an overnight incubation, full media was replaced with assay DMEM media (described above) and cells were incubated in a 37 °C incubator for 24 hours. Cell were then treated with 4 µM P259 for 15 minutes. Following treatment, imaging was performed live on a Leica DMI6000B inverted microscope. All mitochondria were imaged with a GFP filter. Then, green dendra fluorescence was converted to red by blue DAPI filtered light at 100x objective with a 1 A.U. pinhole diameter. The photoconverted red signal was then captured using the TRITC red filter every 30 seconds for 5 minutes. The initial image was subtracted from each subsequent image to track signal change over time.

Transgenic expression of P259

In order to tandemly express P259 and GFP in mammalian cells, five-prime phosphorylated primers were designed to insert the P259 peptide sequence downstream of the CMV promoter of pSF-CMV-PGK-eGFP dual promoter GFP plasmid (Oxford Genetics, Oxford, UK). The primers were used to amplify the vector template and the linearized template was ligated with T4 DNA Ligase (New England Biolabs, MA, USA). Ligated vector was transformed and amplified in TOP10 competent cells (Thermo Fisher, MA, USA). Alanine substitutions were introduced into the GFP-P259 vector by QuickChange II (Agilent, CA, USA) as described by the manufacturer.

SH-SY5Y neuroblastoma cells were seeded in a 96-well plate at 15,000 cells/well overnight. Cells were transfected with 100 ng of the GFP-P259 vector using 100 ng of lipofectamine 2000 (ThermoFisher, MA, USA). After 8 hours of incubation at 37 °C, culture media was replaced. After two days of incubation at 37 °C, mitochondrial ROS was measured in each cell as described above, adding a fluorescence image corresponding to peptide expression with a GFP filter (Keyence). Using MATLAB, single nuclei were segmented. The GFP and MitoSox signals were measured in a ring around each nucleus. Average MitoSox levels were quantified in each GFP-positive cell. The average Mitosox intensities were normalized to the GFP-empty vector.

Oxygen consumption rate

SH-SY5Y cells were seeded overnight in a 24-well (Seahorse Bioscience) plate at 40,000 cells/well. Cells were then treated overnight with the indicated amounts of peptide. Following overnight treatment, the growth medium was replaced with 575µl of bicarbonate-free basal media at pH 7.4 (Seahorse Bioscience) for 1 hour at 37 °C prior to measuring basal oxygen consumption rate (OCR) and extracellular acidification rate (ECAR) using XFe24 Extracellular Flux Analyzer (Seahorse Bioscience).

After attaining baseline measurements, 75µl of oligomycin, 75µl of FCCP, 75µl of rotenone/ antimycin-A was sequentially added to each well to assess OCR and ECAR in different conditions. Changes in oxygen consumption in different conditions were utilized to calculate ATP linked oxygen consumption, maximum respiratory capacity, reserve capacity and non-mitochondrial respiration.

Mitochondrial motility

Primary cortical neurons cultured for 10 days as described above were stained with 50 nM TMRM for 1 hour in the presence of 4 µM P259 or a no peptide control. Following the incubation, mitochondria were imaged through a TRITC filter with a 100X oil immersion objective on a BZ-

X700 microscope equipped with a temperature and CO_2 regulation chamber (Keyence). The images were obtained live at the rate of 1 image every 30 seconds for a total of 20 minutes. To prevent bleaching, only 0.3% of the laser power was utilized.

To quantify mitochondrial motility, the images at time 0 and 150 seconds were overlaid. For each pixel, the intensity at each time was plotted using the bivariate kernel density estimate (kdeplot) feature in the seaborn statistical visualization package for Python. A perfect correlation between time 0 and 150 seconds would correspond to no movement, while a random distribution of pixels would correspond to 100% mobile mitochondria. A Pearson correlation coefficient (PCC) between the two time-points was used to estimate the percentage of mobile mitochondria with the assumption that a PCC value of 1 corresponds to no mobile mitochondrial while a PCC value of 0.5 corresponds to 100% mobile mitochondria.

Peptide treatment in mice

All the experiments were in accordance with protocols approved by the Institutional Animal care and Use Committee of Stanford University and were performed based on the National Institutes of Health Guide for the Care and Use of Laboratory Animals. Five-week old R6/2 HD mice and their wild-type littermates were purchased from The Jackson Laboratory. The mice were housed three to five per cage and handled by a blinded handler for 5 days prior to each behavioral experiment. The mice were provided with ad libitum water and food as wet chow at the bottom of the cage, as the disease affected their ability to reach the food in the hamper. Due to their higher sensitivity to noise and vibration, R6/2 cages were always hand-carried.[10] A total of 60 mice were implanted subcutaneously with a 28-d Alzet osmotic pump (DURECT Co., Cupertino, CA) with either P259-TAT or TAT control peptide (15 mice per group) to be delivered continuously at a rate of 3 mg/Kg/day, as previously described.[10] All mice were implanted twice for a total of 8-week treatment. 20 additional WT mice were implanted 3-times for a total of 12-week treatment at 3 mg/Kg/day.

Isolation of brain mitochondria

The mitochondria fraction from brain tissue was isolated as previously described.[2] Briefly, brain tissue was homogenized in a mannitol-sucrose (MS) buffer (210 mM mannitol, 70 mM sucrose, 5 mM MOPS, 1 mM EDTA, and protease inhibitor cocktail at pH 7.4). Following centrifugation at 800g for 10 minutes, the supernatant was further centrifuged for 15 minutes to isolate the mitochondrial pellet from the cytoplasmic supernatant. The mitochondrial pellet was resuspended in the MS buffer.

ATP content measurement in brain tissue

A ~100 mg sample of flash-frozen brain tissue was weighed and the total ATP content was measured using an ATP colorimetric/fluorometric assay kit (BioVision) according to the manufacturer's instructions. ATP values were normalized to original tissue weight.

Transmission electron microscopy

Sections of the striatum from each mouse brain were fixed in 2% glutaraldehyde at 4% formaldehyde in 0.1 M sodium cacodylate at pH 7.4 for 15 minutes at room temperature and then stored overnight at 4 °C. Samples were then further processed by the Stanford Electron Microscopy

Facility as previously described.[11] Images were acquired using a JEOL1400 transmission electron microscope at 500X for quantification and 10,000X for representation. Mitochondrial area was manually traced and quantified using ImageJ software.

Survival and behavioral mice studies

Survival during the study period was recorded and the remaining of the HD R6/2 mice were sacrificed at 13 weeks along with the 5 median mice from the WT TAT and P259 groups (median mice have aggregate behavioral scores close to the median value of each group). All behavior and survival tests were conducted by an experienced observer who was blinded to the treatment groups. Behavioral tests were administered weekly in an open-field activity arena (Med Associates Inc, St. Albans, VT. Model ENV-515) equipped with three planes of infrared detectors within a sound-proof chamber (Med Associates Inc., St. Albans, VT. MED-017-027), as described previously.[12] Each mouse was placed in a corner of the chamber and was tracked for a period of 15 minutes. The following activities were recorded: digging duration, grooming duration, climbing duration, rearing duration, seizure frequency, average velocity, active behaviors, total distance moved, jump counts/time, ambulatory counts/distance/time, latency towards center, stereotypic time/counts, resting time, vertical counts/time, and zone entries (center/periphery).

Dimensionality reduction of mouse behavior data

The collection of weekly behavior parameters for wild-type mice treated with P259 or the TAT control was assembled into a vector with a total of 180 behavior parameters per mouse. The top 20% of parameters contributing to treatment classification were filtered by using a combination of the ANOVA F- and the mutual information scores for each behavior. The filtered dataset was normalized and principal component analysis was performed using the Python scikit-learn package. An eigenvector was extracted to determine the contribution of each behavior to the first principal component.

To compare P259-treated wild-type mice to R6/2 mice, behaviors from P259/control-treated wild-type mice and control-treated R6/2 mice during the eight-week treatment were assembled into a single vector for each mouse. The dataset was normalized and local linear embedding from the Python scikit-learn package was used to reduce the dimensionality of and visualize the behavior parameters.

Supplementary References

1 Choi, Y. & Chan, A. P. PROVEAN web server: a tool to predict the functional effect of amino acid substitutions and indels. *Bioinformatics* **31**, 2745-2747 (2015).

2 Hwang, S., Disatnik, M.-H. & Mochly-Rosen, D. Impaired GAPDH-induced mitophagy contributes to the pathology of Huntington's disease. *EMBO Molecular Medicine* **7**, 1307-1326 (2015).

3 Shamloo, M. *et al.* Death-associated Protein Kinase Is Activated by Dephosphorylation in Response to Cerebral Ischemia. *Journal of Biol Chem* **280**, 42290-42299 (2005).

4 Souroujon, M. C. & Mochly-Rosen, D. Peptide modulators of protein-protein interactions in intracellular signaling. *Nature Biotechnology* **16**, 919-924 (1998).

5 Huang, X. & Miller, W. A time-efficient, linear-space local similarity algorithm. *Advances in Applied Mathematics* **12**, 337-357 (1991).

6 NCBI, RC. Database Resources of the National Center for Biotechnology Information. *Nucleic Acids Res* **45**, D7-D19, https://doi.org/10.1093/nar/gkw1071 (2017).

7 Cock, P. J. A. *et al.* Biopython: freely available Python tools for computational molecular biology and bioinformatics. *Bioinformatics* **25**, 1422-1423 (2009).

8 Zerbino, D. R. *et al.* Ensembl 2018. *Nucleic Acids Research* **46**, D754-D761, https://doi.org/10.1093/nar/gkx1098 (2018).

9 Qi, X., Qvit, N., Su, Y.-C. & Mochly-Rosen, D. A novel Drp1 inhibitor diminishes aberrant mitochondrial fission and neurotoxicity. *Journal of Cell Science* **126**, 789-802 (2013).

10 Disatnik, M.-H. *et al.* Potential biomarkers to follow the progression and treatment response of Huntington’s disease. *The Journal of Experimental Medicine* **213**, 2655-2669 (2016).

11 Yogalingam, G., Hwang, S., Ferreira, J. C. & Mochly-Rosen, D. Glyceraldehyde-3-phosphate dehydrogenase (GAPDH) phosphorylation by protein kinase Cdelta (PKCdelta) inhibits mitochondria elimination by lysosomal-like structures following ischemia and reoxygenation-induced injury. *J Biol Chem* **288**, 18947-18960 (2013).

12 Joshi, A. U. *et al.* Inhibition of Drp1/Fis1 interaction slows progression of amyotrophic lateral sclerosis. *EMBO Molecular Medicine* **10**, e8166, https://doi.org/10.15252/emmm.201708166 (2018).
